# Iron Chelator VLX600 Inhibits Mitochondrial Respiration and Promotes Sensitization of Neuroblastoma Cells in Nutrition-Restricted Conditions

**DOI:** 10.3390/cancers14133225

**Published:** 2022-06-30

**Authors:** Amanda Westergren Jakobsson, Snehangshu Kundu, Jing Guo, Azazul Chowdhury, Miao Zhao, Emma Lindell, Peter Bergsten, Fredrik J. Swartling, Tobias Sjöblom, Xiaonan Zhang

**Affiliations:** 1 Department of Immunology, Genetics and Pathology, Uppsala University, SE-751 85 Uppsala, Sweden; amanda.westergrenjakobsson.8216@student.uu.se (A.W.J.); snehangshu.kundu@igp.uu.se (S.K.); miao.zhao@igp.uu.se (M.Z.); emma.lindell.6015@student.uu.se (E.L.); fredrik.swartling@igp.uu.se (F.J.S.); tobias.sjoblom@igp.uu.se (T.S.); 2 Centre for Computational Biology, Duke-NUS Medical School, 8 College Road, Singapore 169857, Singapore; jing.guo@duke-nus.edu.sg; 3 Department of Medical Cell Biology, Uppsala University, BMC, Husargatan 3, SE-75123 Uppsala, Sweden; azazul.chowdhury@mcb.uu.se (A.C.); peter.bergsten@mcb.uu.se (P.B.)

**Keywords:** neuroblastoma, mitochondria, MYCN, mTOR, spheroids, new therapeutic approach

## Abstract

**Simple Summary:**

MYC proteins are essential regulators which could affect more than 15% of all genes through an interaction with other transcription factors. High-risk neuroblastoma associated with treatment failure is characterized by amplification of the MYCN proto-oncogene. Here, we show for the first time that the iron chelator VLX600 inhibits mitochondrial activity and induces cell death, regardless of MYCN status in neuroblastoma cells. Blocking glucose uptake enhances the effect of VLX600, indicating that targeting pathways or cellular activities related to energy supply/metabolism may help to find better therapeutic strategy for neuroblastoma.

**Abstract:**

Neuroblastoma, the most common solid tumor in children, is characterized by amplification of the *MYCN* proto-oncogene, a high-risk aggressive clinical marker associated with treatment failure. MYCN plays an important role in cell growth, proliferation, metabolism, and chemoresistance. Here, we show for the first time that in neuroblastoma, iron chelator VLX600 inhibits mitochondrial respiration, decreases expression levels of MYCN/LMO1, and induces an efficient cell death regardless of MYCN status in both 2D and 3D culture conditions. Moreover, insufficient induction of autophagy was observed in cells treated with VLX600, which is essential as a protective response in the event of ATP synthesis disruption. Further inhibition of glucose uptake using DRB18, a pan-GLUT (glucose transporter) inhibitor, synergized the effect of VLX600 and no significant cell death was found in immortalized epithelial cells under this combination treatment. Our results demonstrate that inhibition of mitochondrial respiration by iron chelator VLX600 accompanied by autophagy deficiency promotes sensitivity of neuroblastoma cells in a nutrition-restricted microenvironment regardless of MYCN status, indicating that MYCN expression level is an essential clinical marker but might not be a necessary target for the treatment of neuroblastoma which warrants further investigation. VLX600 has been studied in Phase I clinical trials; combining VLX600 with conventional chemotherapy could be an innovative therapeutic strategy for neuroblastoma.

## 1. Introduction

Neuroblastoma is the most common solid tumor in children [1]. It is characterized by clinical heterogeneity, ranging from extensive tumor regression or differentiation without any treatment, to multimodal therapy-resistant tumors with metastatic spread [2]. Unfortunately, it is ultimately fatal for most patients if they develop locally aggressive and/or metastatic disease over 1 year of age [3]. The inactivation of tumor suppressor genes and the overexpression of oncogenes are the major somatic genetic contributions to neuroblastoma development [4]. In childhood neuroblastoma, the most important feature appears to be amplification of the *MYCN* proto-oncogene, which occurs in ~25% of primary tumors and is considered a high-risk aggressive clinical marker correlated with advanced stage disease and treatment failure [5,6]. It has been noticed that patients lacking *MYCN* gene amplification will never progress to high-risk disease [6,7,8]. On the contrary, despite the developed strategy of risk-stratified treatments (including radiation, surgery, and chemotherapy), the overall survival for children harboring *MYCN* amplification remains at less than 50% [1,6]. Innovative medicine and strategies are still urgently needed to improve the outcome and survival rate in children with neuroblastoma.

*MYCN* belongs to the small MYC gene family which as well contains *C-MYC* and *L-MYC*. MYC proteins are essential regulators which could affect more than 15% of all genes involved in cell proliferation, growth, metabolism, differentiation, and apoptosis, through an interaction with other transcription factors [9,10]. Studies in mice have indicated that *C-MYC* expresses throughout the mouse embryo at all analyzed developmental stages; however, *MYCN* expression is restricted to hematopoietic stem cells and cells within the developing nervous system [11,12]. This restricted expression pattern of *MYCN* observed in mice supports the clinical observation that neural tumors frequently overexpress *MYCN*, indicating that protooncogene *MYCN* plays an essential role in the development of children neuroblastoma.

Since the MYCN protein has not been directly druggable, an indirect inhibition of MYCN expression by targeting upstream or downstream components of the MYCN pathway is considered an alternative therapeutic option [13,14,15,16]. Oliynyk et al. showed that *MYCN* induction upregulates the activity of glycolytic enzymes and mitochondrial oxidative phosphorylation in neuroblastoma [17], indicating that *MYCN* amplified childhood neuroblastoma could be more dependent on functional mitochondria. In addition, several studies proved that inhibiting mTOR signaling to further downregulate cellular protein synthesis could deregulate *MYCN*-driven proliferation [14,18,19], suggesting that mTOR pathway is another key factor in *MYCN*-amplified children neuroblastoma.

Here, we first time show that in neuroblastoma cells iron chelator VLX600 inhibits mitochondrial respiration, decreases expression levels of MYCN/LMO1 (LIM domain only 1), and induces an efficient cell death regardless of MYCN status. Furthermore, insufficient induction of autophagy is observed in cells treated with VLX600 and further inhibition of glucose uptake using DRB18, a pan-GLUT inhibitor enhances the effect of VLX600. Since VLX600 has been studied in Phase I trials, the combination of VLX600 with conventional chemotherapy may be an attractive treatment strategy for neuroblastoma and warrants further investigation. At the same time, our data may as well indicate that the MYCN expression level is an essential clinical marker but might not be a necessary target for the treatment of neuroblastoma.

## 2. Materials and Methods

### 2.1. Chemicals and Antibodies

VLX600 (S8943), DRB18 (S9636), BEZ-235 (S1009) and chloroquine (S6999) were obtained from Selleck Chemicals (Houston, TX, USA). *MYCN*-amplified neuroblastoma cell lines IMR-32 (Cat. #CCL-127), Sk-N-BE(2) (Cat. #CRL-2271), CHP-212 (Cat. #CRL-2273) and *MYCN*-nonamplified cell lines SH-SY5Y (Cat. #CRL-2266), Sk-N-AS (Cat. #CRL-2137) were obtained from American Type Culture Collection (ATCC). Primary antibodies used for immunoblotting were the following: mouse anti-MYCN (1:1000, abcam, Cambridge, UK, Cat. #ab16898), rabbit anti-LMO1 (1:1000, Invitrogen, Waltham, MA, USA, Cat. #PA5-77246), rabbit anti-4EBP1 (1:1000, Cell Signaling, Denvers, MA, USA, Cat. #9452), rabbit anti-phospho-4EBP1 (1:1000, Cell Signaling, Cat. #2855), mouse anti-actin (1:5000, Santa Cruz Biotechnology, Dallas, TX, USA, Cat. #sc-47778). LC3A/B (1:1000, Cell Signaling Cat. #12741) and MT-COXIV (1:1000, Cell Signaling, Cat. #4850).

### 2.2. Cell Culture

IMR-32 (Cat. #CCL-127) cells were cultured in EMEM (ATCC, Manassas, VA, USA, Cat. #30-2003), supplemented with 10% FBS, 1% non-essential amino acids, and 1% penicillin-streptomycin. Sk-N-BE(2) (Cat. #CRL-2271), CHP-212 (Cat. #CRL-2273) and SH-SY5Y (Cat. #CRL-2266) cells were cultured in a 1:1 ratio of EMEM (ATCC, Manassas, VA, USA, Cat. #30-2003) and Nutrient Mixture F-12, supplemented with 10% FBS, 1% non-essential amino acids, and 1% penicillin-streptomycin. Sk-N-AS (Cat. #CRL-2137), HO15.19 and HO-MYC3 cells were cultured in DMEM medium supplemented with 10% FBS, 1% non-essential amino acids, and 1% penicillin-streptomycin. All cell lines were authenticated by STR profiling (ATCC cell authentication service) and regularly checked for mycoplasma infection with the MycoAlert mycoplasma detection kit (Lonza, Basel, Switzerland).

### 2.3. RNA-SEQ Dataset Analysis

**Figure 1:**
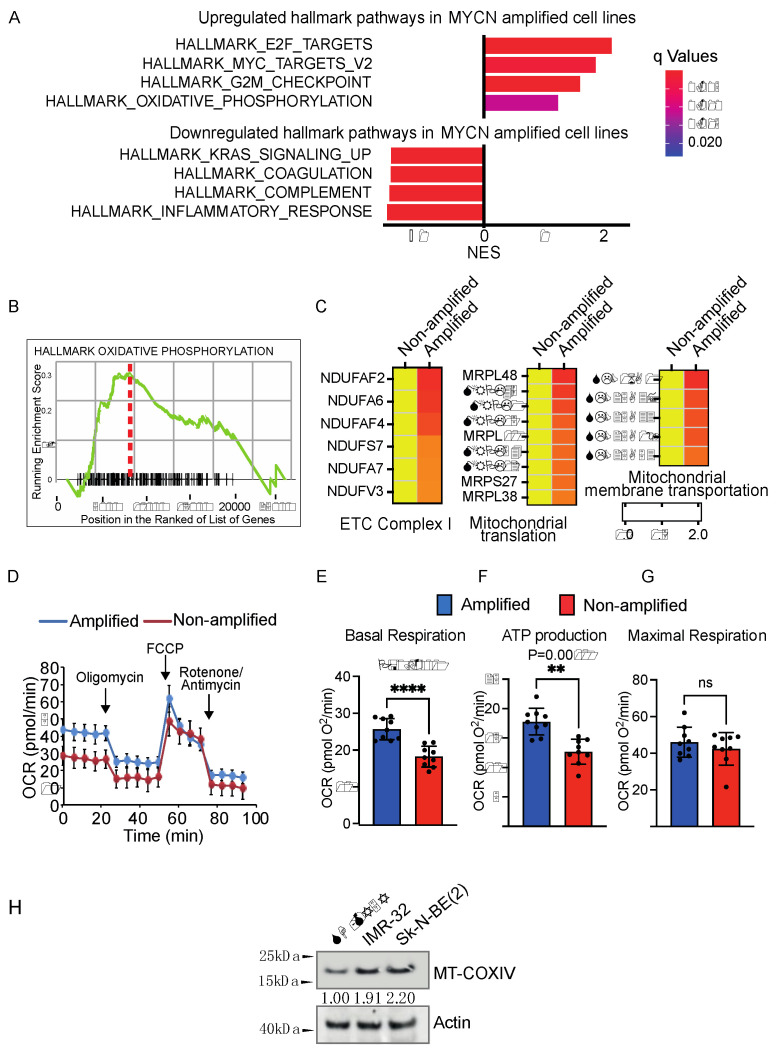
*MYCN*-amplified neuroblastoma cells upregulated the OXPHOS hallmark pathway and mitochondrial activity. (**A**) GSEA data analysis of RNA-SEQ from 39 neuroblastoma cell lines revealed that the OXPHOS (Oxidative phosphorylation) hallmark pathway is among the most upregulated pathways in *MYCN*-amplified neuroblastoma cells. NES: normalized enrichment scores. (**B**) Representative graph for enriched mitochondrial OXPHOS pathway (*p* = 0.008 q = 0.011). (**C**) Genes coding for mitochondrial electron transport chains (ETC), components of mitochondrial translation and transportation machinery are upregulated in *MYCN*-amplified neuroblastoma cells. The full list of changes of mitochondrial genes is included in Supplementary Data S1. (**D**) Oxygen Consumption Rate (OCR) in *MYCN*-amplified Sk-N-BE(2) cells and non-amplified SH-SY5Y neuroblastoma cells. Oligomycin (1.5 μM), FCCP (1.0 μM), antimycin A (0.5 μM), and rotenone (1.0 μM). (**E**–**G**) Basal respiration, ATP-linked respiration and Maximal respiration analyzed from Figure 1D in *MYCN*-amplified and non-amplified neuroblastoma cells. Data are shown as mean ± SD, statistical analysis using two-tailed unpaired *t*-test. ** *p* < 0.01 and **** *p* < 0.0001 and ns: not significant. (**H**) Expression levels of mitochondrial mass marker MT-COXIV in neuroblastoma cells. Here, Sk-N-BE(2) and IMR-32 are *MYCN*-amplified cells and SH-SY5Y are *MYCN*-nonamplified cells. Actin is loading control.

Raw counts from RNA sequencing of the 39 neuroblastoma cell lines were kindly provided by author [20]. Samples were divided into two groups (“high” and “low”) by their validated *MYCN* amplification status reported in the original publication. Samples with FPKM <30 are classified as the MYCN-low group, while FPKM >150 are grouped as the MYCN-high group (FPKM: Fragments Per Kilobase of transcript per Million mapped reads). The cell lines with mid-level MYCN expression, i.e., NBL-S, NLF, NB-16, COG-N-557, COG-N-496, NB-1691, COG-N-471, were not included in the downstream analysis, leaving 35 samples in use. Raw counts of RNA-seq data were processed by DESeq2 [21] version 1.30.1. Differential expression test between the “low” and “high” groups was performed using results function with parameters alpha = 0.05, cooks Cut off = F. Genes are ranked by FDR (False Discovery Rate) of the differential expression. For the functional interpretation of the differential expression test, Gene Set Enrichment test (GSEA) on the MSigDB hallmark gene set [22] are performed using cluster Profiler [23] (version 3.18.1) GSEA function with parameters min GSSize = 5, *p*-value Cut off = 0.05. Names of the gene sets were listed in Figure 1 (The uncropped Western blots have been shown in Figure S3).

### 2.4. Measurements of Oxygen Consumption

The Seahorse XF analyzer was used as recommended by the manufacturer (Seahorse Bioscience, North Billerica, MA, USA). Seahorse Cell Mito Stress Test Kit (Agilent Technologies, Santa Clara, CA, USA) was used to measure mitochondrial OXPHOS. Briefly, 40,000 cells per well were plated in 100 μL culture medium in XF96-well cell plates with blank control wells. Before the measurements of OCR and ECAR, the medium was replaced with 100 μL Seahorse assay media at 37 °C without CO_2_ for 1 h. In order to investigate the effect of VLX600 on mitochondrial function in NB cells, after equilibration at 37 °C without CO_2_ for 1 h, plates were read using protocol as described:

Measure basal OCR and ECAR level 3 times
Inject VLX600 through port A
Measure OCR and ECAR level at indicated times
Inject port B, oligomycin, Measure OCR level 3 times
Inject port C, FCCP, Measure OCR level 3 times
Inject port D, antimycin A/rotenone, Measure OCR level 3 times

In this paper, we used 1.5 μM oligomycin, 1.0 μM FCCP, 0.5 μM antimycin A, and 1.0 μM rotenone. ATP linked respiration OCR were calculated as average values of basal OCR minus average postoligomycin OCR values. Maximum mitochondrial respiration capacity was calculated as average maximal OCR values minus average postantimycin A/rotenone OCR values.

### 2.5. Western Blot

Cells were collected and lysed in ice-cold RIPA buffer (with proteinase and phosphor inhibitor) and run on NuPAGE™ 4 to 12% Bis-Tris Mini Protein Gels (Invitrogen) and transferred to nitrocellulose membranes using the iBlot^®^ 7-Minute Blotting System (Invitrogen). Membranes were pre-treated with SuperSignal^®^ Western Blot Enhancer Antigen Pretreatment Solution (Thermo Scientific, Waltham, MA, USA) for 10 min, washed according to the manufacturer’s protocol, and subsequently blocked with 5% BSA in 1X PBST for 1 h. The membranes were probed and incubated overnight using the following antibodies in 5% BSA in 1X PBST: mouse anti-MYCN (1:1000, abcam, Cat. #ab16898), rabbit anti-LMO1 (1:1000, Invitrogen, Cat. #PA5-77246), rabbit anti-4EBP1 (1:1000, Cell Signaling, Cat. #9452), rabbit anti-phospho-4EBP1 (1:1000, Cell Signaling, Cat. #2855), mouse anti-actin (1:5000, Santa Cruz Biotechnology, Cat. #sc-47778), LC3A/B (1:1000, Cell Signaling Cat. #12741) and MT-COXIV (1:1000, Cell Signaling, Cat. #4850). The next day, membranes were washed with 1xPBST buffer and incubated with anti-rabbit or anti-mouse secondary antibodies at a 1:5000 dilution for 1 h. SuperSignal^®^ West Femto Maximum Sensitivity Substrate (Cat. #9452, Thermo Scientific) was used to detect the signal using Amersham Imager 680 (GE Healthcare, Chicago, IL, USA).

### 2.6. qPCR Analysis for Expressions of MYCN and LMO1 Transcripts

The cDNAs were prepared directly from cell lysates using the TaqMan^TM^ Fast Advanced Cells-to-CT^TM^ Kit (Thermo Fisher Scientific, A35377). The expressions of *MYCN* and *LMO1* transcripts were quantified by RT-qPCR using TaqMan probes against *MYCN* (Hs00232074_m1) and *LMO1* (Hs04487089_m1), respectively, using *ACTB* (Hs01060665_g1) as a reference control. A non-parametric Mann–Whitney test was applied to test the significance level in GraphPad Prism9.

### 2.7. Cell Viability Assay

Cell viability assay was performed using resazurin-based cell viability assay. To perform the resazurin-based cell viability assay, cells were plated in a 96-well plate at a density of 15,000 cells per well. Plated cells were then incubated for 24 h before the treatment. After treatments, cells were incubated for 72 h at 37 °C. Medium was removed, then cells were incubated with resazurin (Sigma-Aldrich, St. Louis, MO, USA) solution for 2.5 h at 37 °C and the fluorescence intensity was read using microplate reader Victor2 1420 multilabel counter (Wallac, Mt. Waverley, Australia) at 530 nm excitation/590 nm emission filter set. The percentage of cell viability was calculated as (fluorescence intensity of treated cells − fluorescence intensity of background)/(fluorescence intensity of DMSO treated control − fluorescence intensity of background) × 100%. The half-maximal inhibitory concentration (IC_50_) was calculated from three individual dose-response experiments using nonlinear regression, dose-response-inhibition analysis in GraphPad prism 9.

### 2.8. Generation of Spheroids

For Incucyte-based image acquisition, a cell suspension containing 1000 cells/50 μL was added to each well of the 384-well plates (Corning, Corning, NY, USA, # 4516). Plates were incubated for 4 days before drug exposure. Then, another 50 μL medium containing 2X VLX600 concentration was added to each well to generate 1X indicated VLX600 concentration. Images of spheroids were taken by Incucyte camera and diameters were then measured and recorded using Incucyte software. Dead cells separate from the spheroids. The circles (white) indicated the remaining part of the spheroids. The volume of the spheroids was calculated by the equation: Volume = 4/3 × 3.14 × R^3^ (R = radius).

For immunoblotting analysis and hematoxylin staining, spheroids were prepared using our previously described method [17]. 15,000 cells/well were plated in 96-well plates (Corning, # 7007). Plates were incubated for 4 days before drug exposure. Spheroids were then treated with indicated concentrations of VLX600 for 24 h and followed with the process of immunoblotting analysis, or spheroids were then treated with VLX600 for 72 h and followed with the process of hematoxylin staining.

### 2.9. Colony Formation Assay

Medium was removed from wells after 3-days long drug exposure. Then, the spheroids were dissociated in 50 μL trypsin, incubated at 37 °C, pipette intervals until the spheroids were dissociated. Cell suspension from each 384-well was accordingly transferred into 6-well plates containing 5 mL medium in each well. Cells in a 6-well plate were further cultured for another 10 days. The medium was changed every 3 days.

### 2.10. Crystal Violet Staining

Cells were fixed with ice cold methanol for 15 min, then methanol was removed and crystal violet staining solution (0.5 g crystal violet powder in 80 mL distilled H_2_O and 20 mL methanol) was added and plates were incubated for 30 min (the volume should cover the whole surface of each well). Then, staining solution was removed and plates were washed with water three times. Let the plates dry at room temperate.

### 2.11. Synergy Effect Analysis

Cells were treated with different concentrations of VLX600 and DRB18 and the viability was determined using a resazurin-based assay. The coefficient of drug interaction (CDI) was used to analyze the interactions between VLX600 and GLUT inhibitor DRB18. According to CDI values, the interactions were categorized as synergism, additivity or antagonism, respectively. CDI was calculated as follows:

CDI = AB/(A × B)

where:

AB = cell viability value for the combination of the VLX600 and DRB18.
A and B = cell viability value for the single treatment VLX600 or DRB18.

A CDI value <1, =1 or >1 indicates that the drugs are synergistic, additive or antagonistic, respectively. A CDI value less than 0.7 indicates that the drugs are significantly synergistic [24].

## 3. Results

### 3.1. MYCN-Amplified Neuroblastoma Cells Upregulated the OXPHOS Hallmark Pathway and Mitochondrial Activity

*C-MYC* upregulation has been reported to enhance mitochondrial activity in various tumors [25,26,27,28]; since *MYCN* and *C-MYC* belong to the same gene family, we speculated that mitochondrial activity could be as well strengthened in *MYCN*-amplified neuroblastoma cells. In order to confirm this hypothesis, we firstly reanalyzed an RNA-SEQ dataset from 39 neuroblastoma cell lines using the same definition for *MYCN*-amplified and *MYCN*-nonamplified groups described in the study [20]. Interestingly, gene set enrichment analysis (GSEA) revealed OXPHOS (oxidative phosphorylation) as the most prominently upregulated hallmark pathway in *MYCN*-amplified neuroblastoma cells, which is associated with an upregulation of the MYC-targeted pathway (Figure 1A,B). Next, we filtered all the mitochondrial genes included in the human mitochondrial MitoCarta2.0 dataset [29] and selected mitochondrial genes which have ≥1.5 expression fold change in the *MYCN*-amplified group comparing with that in the *MYCN*-nonamplified group (Supplementary Data S1). Intriguingly, we observed an enrichment in mitochondrial genes encoding complex I of the mitochondrial electron transport chain (ETC), components of mitochondrial translation and transportation machinery (Figure 1C). To further validate the finding obtained from RNA-SEQ of 39 neuroblastoma cell lines, we measured the mitochondrial O_2_ consumption rates (OCR) at the basal state, after oligomycin (an inhibitor of ATP synthesis), carbonyl cyanide-4 (trifluoromethoxy) phenylhydrazone (FCCP; uncouples ATP synthesis from the ETC), and rotenone and antimycin A, which interfere with complex I and complex III of the ETC, respectively [30] (Figure 1D). We found that basal respiration levels and ATP-linked respiration (Figure 1E,F) were significantly elevated in *MYCN*-amplified cells but the maximal respiration level showed no significant difference between the *MYCN*-amplified and nonamplified cell groups (Figure 1G), indicating that *MYCN*-amplified cells have upregulated mitochondrial activity under normal circumstances but, however, show no superiority under stress conditions [31]. We further checked the mitochondrial mass marker MT-COXIV and noticed an increase (~2-fold change) in *MYCN*-amplified cells, suggesting that the upregulation of mitochondrial O_2_ consumption rates could relate to the raised mitochondrial amount (Figure 1H).

### 3.2. Iron Chelator VLX600 Inhibited Mitochondrial Respiration and Induced Efficient Cell Death in MYCN-Amplified Neuroblastoma Cells

**Figure 2:**
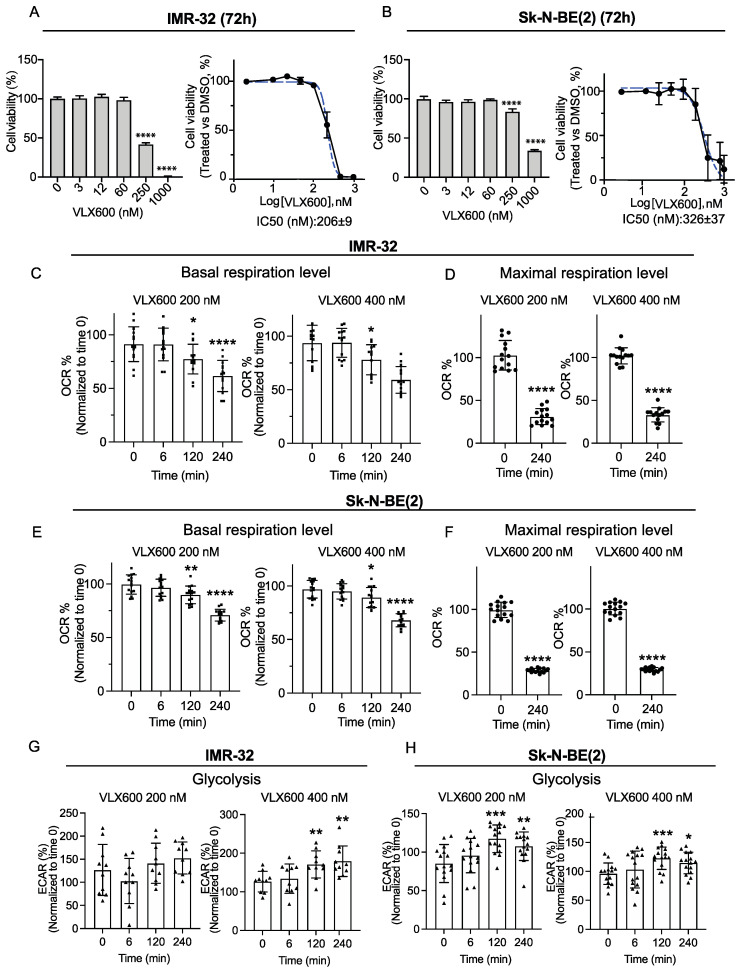
Iron chelator VLX600 inhibited mitochondrial respiration and induced efficient cell death in MYCN-amplified neuroblastoma cells. (**A**,**B**) Dose-dependent sensitivity of *MYCN*-amplified IMR-32 and Sk-N-BE(2) neuroblastoma cells to VLX600. **Left**: bar chart, dose response from an individual experiment. **Right**: dose-response curve from three individual experiments. IC_50_ 206 ± 9 nM in IMR-32 cells and IC_50_ 326 ± 37 nM in Sk-N-BE(2). (**C**) Basal respiration level in IMR-32 cells treated with 200 nM or 400 nM VLX600. Oxygen Consumption Rate (OCR) was measured at indicated time points after VLX600 injection (6, 120 and 240 min). Final data are presented as percentage (%, normalized to values at time 0). (**D**) Maximal respiration level in IMR-32 cells treated with 200 nM or 400 nM VLX600 for 4 h. Oxygen Consumption Rate (OCR) was measured after FCCP injection. Final data are presented as percentage (%, normalized to values at time 0). (**E**) Basal respiration level in Sk-N-BE(2) cells treated with 200 nM or 400 nM VLX600. Oxygen Consumption Rate (OCR) was measured at indicated time points after VLX600 injection (6, 120 and 240 min). (**F**) Maximal respiration level in Sk-N-BE(2) cells treated with 200 nM or 400 nM VLX600 for 4 h. Oxygen Consumption Rate (OCR) was measured after FCCP injection. Final data are presented as percentage (%, normalized to values at time 0). (**G**) Glycolysis in IMR-32 cells treated with 200 nM or 400 nM VLX600. Extracellular acidification rate (ECAR) was measured at indicated time points after VLX600 injection (6, 120 and 240 min). (**H**) Glycolysis in Sk-N-BE(2) cells treated with 200 nM or 400 nM VLX600. Extracellular acidification rate (ECAR) was measured at the indicated time points after VLX600 injection (6, 120 and 240 min). Data are shown as mean ± SD, statistical analysis using two-tailed paired *t*-test. * *p* < 0.05, ** *p* < 0.01, *** *p* < 0.001 and **** *p* < 0.0001.

*MYCN*-amplified neuroblastoma cells showed an elevated ATP demand (Figure 1E,F), indicating that *MYCN*-amplified cells could be more dependent on functional mitochondria, thus leading to a limited toleration to mitochondrial inhibition. Iron chelator VLX600 [32], which has been evaluated in a clinical phase I trial (NCT02222363), was reported to inhibit the complexes I, II and IV on ETC and show superior efficacy on solid tumors [33]. We noticed that *MYCN*-amplified neuroblastoma cells exhibited a significant vulnerability to VLX600, with an IC_50_ 206 ± 9 nM in IMR-32 cells and IC_50_ 326 ± 37 nM in Sk-N-BE(2) cells compared with that in human colon tumor cells (IC_50_ ~ 6.5 µM) (Figure 2A,B). Although VLX600 has been noted to inhibit mitochondrial activity in colon cancer cells at IC_50_ ~ 6.5 µM [33], it remains uncertain whether VLX600 can inhibit mitochondrial respiration in neuroblastoma cells. To confirm this, we chose IC_50_ concentrations and monitored mitochondrial O_2_ consumption rates in IMR-32 and Sk-N-BE(2) cells at the indicated time points. Consistent with our previous observation in colon cancer cells, we also noticed reduced O_2_ consumption rates in IMR-32 and Sk-N-BE(2) cells at VLX600 200 nM and 400 nM (Figure 2C,E). After 4 h of treatment, no stimulation of the O_2_ consumption rate was observed after FCCP (Figure 2D,F), suggesting that VLX600 impairs mitochondrial function in neuroblastoma cells. It is known that inhibition of OXPHOS triggers a mitochondrial metabolism shift to glycolysis to maintain the intracellular ATP level and essential cell functions [34]. Consistent with our previous finding in colon cancer cells [33], we as well noticed an upregulation of glycolysis in both IMR-32 and Sk-N-BE(2) cells at 200 nM and 400 nM (Figure 2G,H).

### 3.3. VLX600 Led to Decreased Spheroid Growth of MYCN-Amplified Neuroblastoma

**Figure 3:**
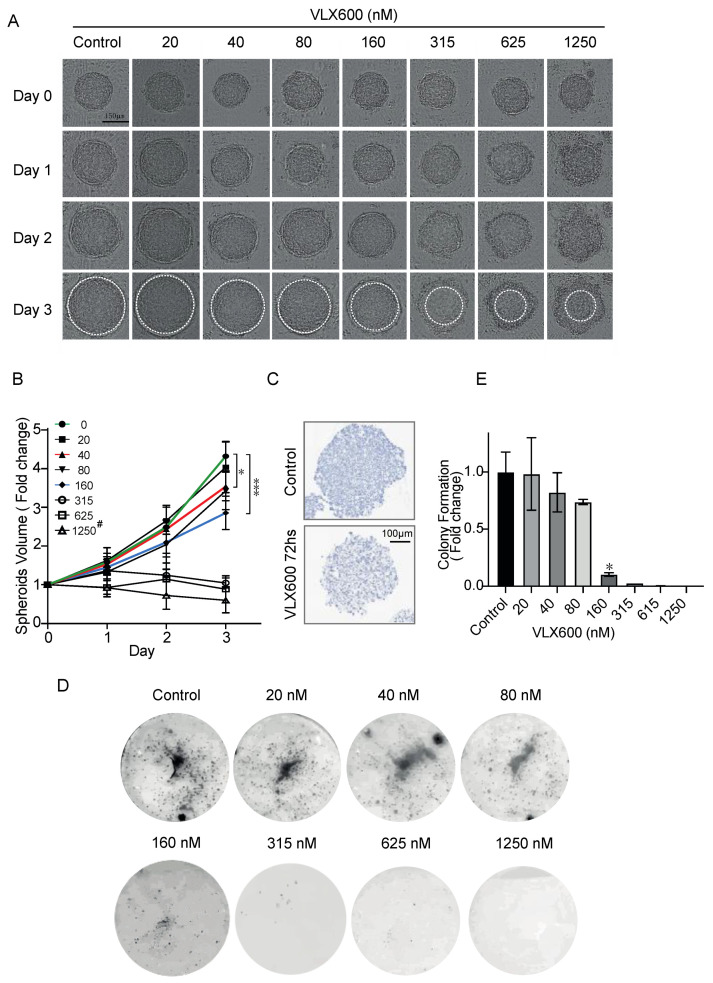
VLX600 led to decreased spheroid growth of MYCN-amplified neuroblastoma. (**A**) IMR-32 derived spheroids exposed to the indicated concentrations of VLX600 for 72 h. Every 24 h, pictures under each condition were recorded (*n* = 5). Dead cells will gradually separate from spheroids after VLX600 treatment. The circles (white) indicate the remaining part of spheroids, assisting for the diameter measurement and spheroids volume calculation. (**B**) Quantification of residual spheroid volume in cells exposed to the indicated concentrations of VLX600. 1250nM^#^: a remaining part of spheroid was still observed in (**A**,**B**), however, no colony was generated under the same condition (**D**), indicating that the assessment of the percentage of viable cells after VLX600 cannot solely depend on the remaining volume of spheroids. (**C**) Hematoxylin staining for spheroids treated with either DMSO or 200 nM VLX600 for 72 h. (**D**) Colony formation assay for 10 days for cells from the spheroids treated with either DMSO and VLX600. (**E**) Quantifications of colony formation assay (*n* = 2, two repeats). The *p* values for statistical significance were determined using a *t*-test, * *p* < 0.05 and *** *p* < 0.001.

In our previous study, a comparable IC_50_ was observed between monolayer and spheroid culture conditions in human colon tumor cells. Thus, we performed spheroid-cultures in *MYCN*-amplified IMR-32 and Sk-N-BE(2) cell lines but Sk-N-BE(2) cells could not form neat spheroids for the following spheroid diameter measurement (Figure S1). We proceeded with spheroid-culture experiments using *MYCN*-amplified IMR-32 cells and spheroids were treated with ascending concentrations of VLX600. It could be clearly seen that spheroids were smaller at concentration 40nm and started to loss the integrity of shape at 160 nM after 72 h, indicating that the cell proliferation starts to be inhibited at lower concentration (40 nM) and cell death is induced at higher concentration (≥160 nM) (Figure 3A). Compared with the spheroids treated with only DMSO, the volume of spheroids at 160 nM was almost 50% smaller after 72 h (*p* < 0.001; *t*-test), revealing that the VLX600 IC_50_ under 3D conditions is relatively similar to that under monolayer 2D conditions (Figure 3B). We further sectioned spheroids treated with DMSO or 200nM VLX600 for 72 h and stained them using hematoxylin. Consistent with the observation from Figure 3A, spheroids treated with VLX600 showed a decreased diameter (~460 µM vs. 380 µM) (Figure 3C). Dead cells will separate from the spheroids. The circles (marked in white in Figure 3A) indicated the remaining part of spheroids. However, the assessment of the percentage of viable cells after VLX600 cannot solely depend on the remaining volume of spheroids. In order to further confirm the effect of VLX600 on spheroids, we trypsinized spheroids into single cells after 72 h of drug exposure and cultured them in 6-well plates for 10 days. Surprisingly, at VLX600 160 nM, only ~10% cells recovered compared with the spheroids treated with DMSO (*p* < 0.5; *t*-test) and higher concentration (≥315 nM) almost deprived the ability of cell re-population (Figure 3D,E), indicating that VLX600 has a strong drug penetration ability, and higher concentrations of VLX600 can prevent the regeneration of spheroids during the drug-free period in vitro.

### 3.4. Mitochondrial Inhibition Decreased the Level of MYCN and Its Associator LMO1

**Figure 4:**
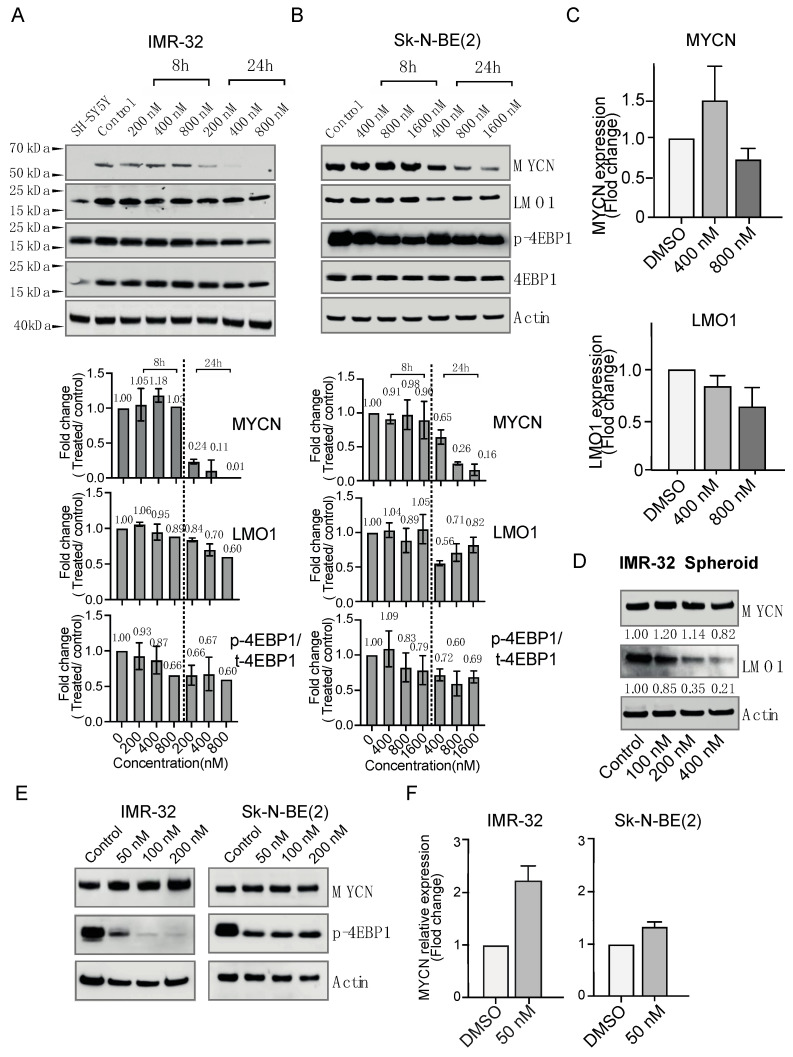
Mitochondrial inhibition decreased the level of MYCN and its associator LMO1. (**A**) (**Upper**) Expression levels of MYCN, LMO1, total-4EBP1 and phospol-4EBP1 in *MYCN*-amplified IMR-32 cells exposed to indicated concentrations of VLX600 for 8 or 24 h. (**Lower**) Quantitative analysis of the indicated band density. (**B**) (**Upper**) Expression levels of MYCN, LMO1, total-4EBP1 and phospol-4EBP1 in *MYCN*-amplified Sk-N-BE(2) cells exposed to indicated concentrations of VLX600 for 8 or 24 h. (**Lower**) Quantitative analysis of the indicated band density. (**C**) mRNA expression levels of *MYCN* and *LMO1* were measured by quantitative PCR (qPCR) in Sk-N-BE(2) cells treated with DMSO or VLX600. (**D**) Expression levels of MYCN and LMO1 in *MYCN*-amplified IMR-32 spheroids after 24-h incubation with VLX600. (**E**) Expression levels of MYCN and phospol-4EBP1 in *MYCN*-amplified IMR-32 and Sk-N-BE(2) cells treated with indicated concentrations of BEZ-235 for 24 h. (**F**) mRNA expression levels of *MYCN* measured by quantitative PCR (qPCR) in IMR-32 and Sk-N-BE(2) cells treated with DMSO or 50 nM BEZ-235.

VLX600 has been reported to decrease the expression level of C-MYC in human colon tumor cells [35]. Since *MYCN* and *C-MYC* belong to a same family, we were wondering if the inhibition of mitochondrial function by VLX600 could also reduce the protein level of MYCN in *MYCN*-amplified neuroblastoma cells. In order to check this hypothesis, we treated *MYCN*-amplified cells with different concentrations of VLX600 for both 8 and 24 h. The results showed that MYCN was significantly decreased (80–90% reduction) after 24 h at IC_50_ concentration. LMO1 is implicated as an independent predictor of poor patient survival in the clinic [36] and its co-expression with MYCN was associated with an earlier onset and increased penetrance of neuroblastoma [37], indicating that the inhibition of MYCN might lead to a concomitant decrease of LMO1. Therefore, we further detected the protein level of LMO1 after the treatment of VLX600. A reduction was indeed observed after 24-h incubation of VLX600 at the indicated concentrations (Figure 4A,B). At the transcript level, we observed a reduction of *MYCN* and *LMO1* at concentration (800 nM), higher than IC_50_ (~400 nM) (Figure 4C). The mTOR signaling can be stimulated by phosphorylating key translation factors (4EBP1/eIF4E) upstream of the translation initiation complex and further stabilizing MYCN protein levels by inducing MYCN translation [19,38]. Since VLX600 was reported to inhibit mTOR signaling resulting from the mitochondrial inhibition-induced energy crisis, we performed an immunoblotting assay to determine the level of p-4EBP1/t-4EBP1 after incubation of VLX600, and we noticed a reduction in the ratio of p-4EBP1/4EBP1 in *MYCN*-amplified neuroblastoma cells (Figure 4A,B), revealing a downregulation of the mTOR pathway. Since we noticed a strong effect of VLX600 on 3D spheroids (Figure 3), we wondered if a reduction on MYCN and LMO1 can be observed under this condition. In order to test this, we treated spheroids with different concentrations of VLX600 for 24 h and proceeded for an immunoblotting analysis. We did notice a strong and moderate level of reductions for LMO1 and MYCN, respectively (Figure 4D), However, a sole inhibition on the mTOR/PI3K pathway with an inhibitor BEZ-235 was not able to significantly reduce the expression of MYCN both at the translation level and transcript level (Figure 4E,F, respectively), suggesting that mitochondrial inhibition is a major cause of MYCN reduction and mTOR is not upstream of MYCN regulation.

### 3.5. VLX600 Induced a Similar Cytotoxicity in MYCN Non-Amplified Neuroblastoma Cells

**Figure 5:**
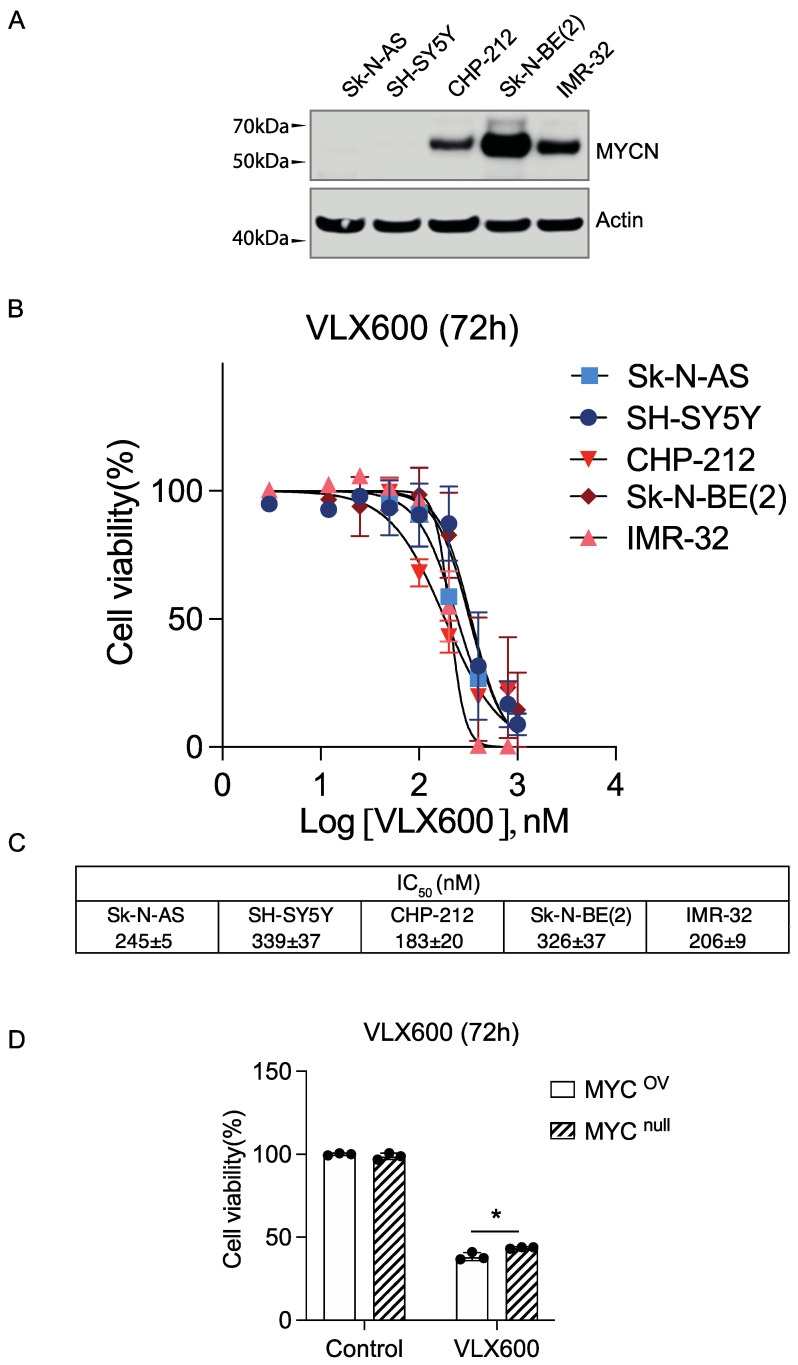
VLX600 induced a similar cytotoxicity in MYCN non-amplified neuroblastoma cells. (**A**) MYCN expression level in five neuroblastoma cell lines (Sk-N-AS, SH-SY5Y, CHP-212, Sk-N-BE(2) and IMR-32), Sk-N-AS and SH-SY5Y are MYCN non-amplified neuroblastoma cells; CHP-212, Sk-N-BE(2) and IMR-32 are MYCN-amplified neuroblastoma cells. (**B**) Dose-dependent response curve of Sk-N-AS, SH-SY5Y, CHP-212, Sk-N-BE(2) and IMR-32 to VLX600 for 72 h. (**C**) IC_50_ calculated from (**B**) for five neuroblastoma cell lines. (**D**) Cell viability after 72 h VLX600 in cell lines including HO15.19 (a MYC null cell line, MYC^null^) and HO-myc3 (a MYC overexpressed cell line, MYC^OV^) which was as well used in our previous paper [35]. Data are shown as mean ± SD, statistical analysis using *t*-test. * *p* < 0.05.

Our previous finding and results from other studies [39,40,41] have shown that cancer cells with an upregulated mitochondrial function are more sensitive to mitochondrial inhibition. Therefore, we speculated that *MYCN* non-amplified neuroblastoma cells would be more resistant to VLX600. In order to test this hypothesis, we included five cell lines (*MYCN* non-amplified neuroblastoma cells: Sk-N-AS and SH-SY5Y; *MYCN*-amplified neuroblastoma cells: CHP-212, Sk-N-BE(2) and IMR-32) (Figure 5A). Unexpectedly, the IC_50_ value of *MYCN* non-amplified and *MYCN*-amplified neuroblastoma cells were relatively comparable (Figure 5B,C). Then, we tested VLX600 in a cell model used in our previous paper [35], including HO15.19 (a MYC null cell line, MYC^null^) and HO-myc3 (a MYC overexpressed cell line, MYC^OV^). The cell viability after 72-h treatment was still comparable, suggesting that overexpression of MYCN/MYC has no effect on VLX600-induced cytotoxicity (Figure 5D). Taken together, our data suggested that mitochondrial inhibitor VLX600 could induce an efficient cell death regardless of MYCN status.

### 3.6. Autophagy as a Rescue Response to VLX600 Was Not Observed in Neuroblastoma Cells

**Figure 6:**
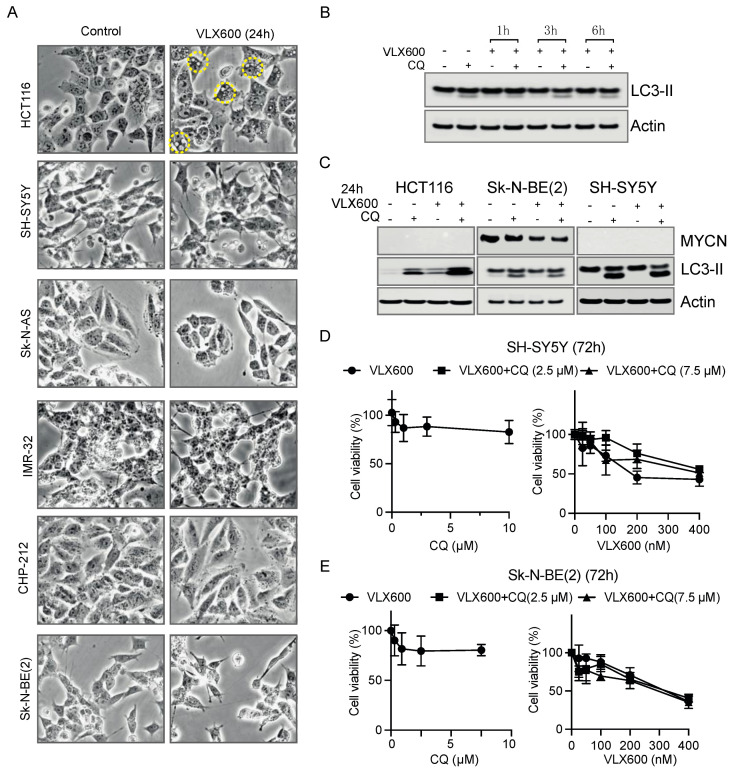
Autophagy as a rescue response to VLX600 was not observed in neuroblastoma cells. (**A**) Images for HCT116, SH-SY5Y, Sk-N-AS, IMR-32, CHP-212 and Sk-N-BE(2) cells treated for DMSO or VLX600 for 24 h. Yellow circles marked in HCT116 cells indicate the large cytoplasmic vesicles. (**B**) Expression levels of LC3-II in Sk-N-BE(2) cells treated with 400 nM VLX600 for indicated hours absent or present CQ. (**C**) Expression levels of MYCN, LC3-II in HCT116, Sk-N-BE(2) and SH-SY5Y cells treated with VLX600 for 24 h absent or present CQ. HCT116 cells only express C-MYC. A further accumulation of LC3-II was noticed in HCT116 cells but not in *MYCN*-amplified Sk-N-BE(2) and *MYCN*-nonamplified SH-SY5Y neuroblastoma cells. (**D**,**E**) *MYCN*-amplified Sk-N-BE(2) and *MYCN*-nonamplified SH-SY5Y cells were treated with VLX600 alone or combined with CQ (2.5 µM or 7.5 µM) and cell viability value was obtained after 72 h. Blocking autophagy by CQ cannot enhance the effect of VLX600 at indicated concentrations.

In our previous study, we noticed a presence of large cytoplasmic vesicles which were stained positively with lysotracker in HCT116 cells; however, these large cytoplasmic vesicles cannot be observed in neuroblastoma cells (Figure 6A). Induction of autophagy was shown to be a protective response to VLX600 in colon cancer cells [33,42]. Therefore, we speculated that neuroblastoma cells cannot adequately induce autophagy when exposed to VLX600. To investigate this possibility, we examined the expression levels of LC3-II (the phosphatidylethanolamine-conjugated form of LC3), an autophagosome marker that has been used to study autophagy [43]. We incubated *MYCN*-amplified Sk-N-BE(2) cells with VLX600 absent or present the lysosomotropic agent chloroquine (CQ) known as an autophagy inhibitor. Under control conditions, when CQ was present, we noticed a slight accumulation of LC3-II, showing basal levels of autophagy in cells. However, we did not notice a further accumulation of LC3-II within 6-h treatment with VLX600 (Figure 6B). Longer treatments over 24 h showed a consistent response to VLX600 in both *MYCN*-amplified and non-amplified cells (Figure 6C), suggesting that autophagy is not properly induced in neuroblastoma cells exposed to VLX600. Moreover, the combination of VLX600 with CQ was not able to generate a synergistic effect over a 72-h incubation (Figure 6D,E). Taken together, our data suggest that autophagy as a rescue response to VLX600 is not properly triggered in neuroblastoma cells.

### 3.7. VLX600 Synergized with GLUT Inhibitor DRB18 in Neuroblastoma Cells

**Figure 7:**
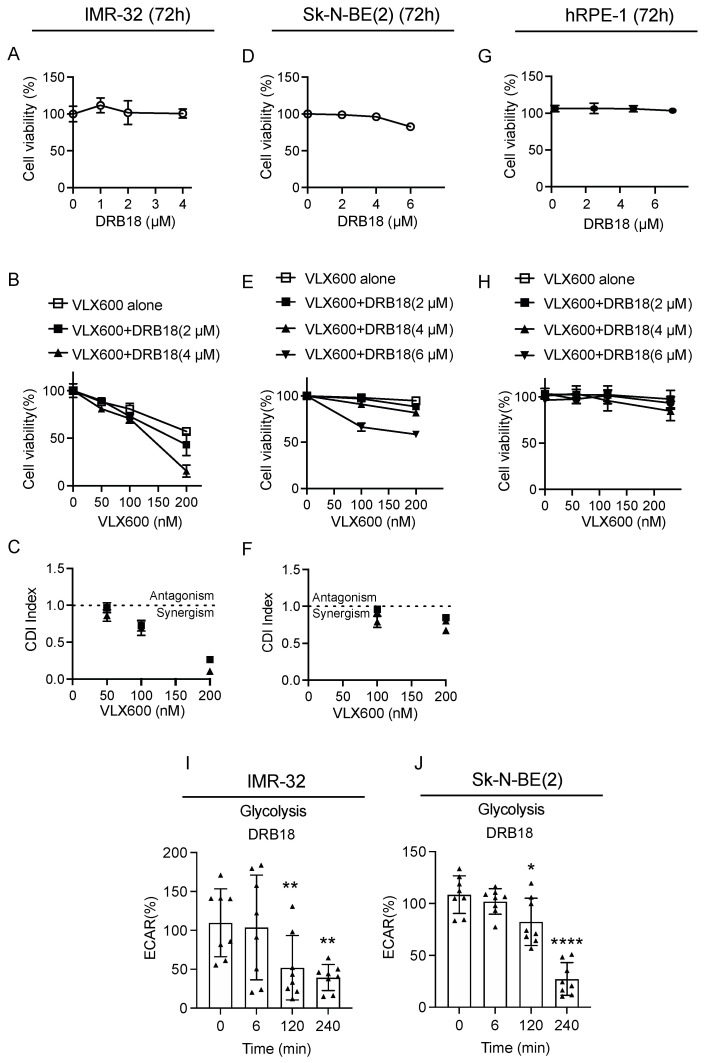
VLX600 synergized with GLUT inhibitor DRB18 in neuroblastoma cells. (**A**,**D**) Concentrations of DRB18 generating minimal cytotoxic effects on IMR-32 and Sk-N-BE(2) cells after 72 h treatment. (**B**,**C**,**E**,**F**) IMR-32 and Sk-N-BE(2) cells were treated with DMSO, VLX600 alone, or VLX600/DRB18 for 72 h. CDI was calculated as follows: CDI = AB/(A × B) where: AB = cell viability value for the combination of the VLX600 and DRB18. A and B = cell viability value for the single treatment VLX600 or DRB18. A CDI value <1, =1 or >1 indicates that the drugs are synergistic, additive or antagonistic, respectively. A CDI value less than 0.7 indicates that the drugs are significantly synergistic [24]. (**G**) Concentrations of DRB18 used in IMR-32 and Sk-N-BE(2) cells did not generate obvious cytotoxicity in hRPE-1 cells. (**H**) hRPE-1 cells treated with DMSO, VLX600 alone, or VLX600/DRB18 for 72 h. Concentrations were consistent with that used in IMR-32 and Sk-N-BE(2) cells and no obvious cytotoxicity was observed in hRPE-1 cells. (**I**,**J**) Glycolysis in IMR-32 and Sk-N-BE(2) cells treated with 4 µM DRB18. Extracellular acidification rate (ECAR) was measured at indicated time points after DRB18 injection (6, 120 and 240 min). Data are shown as mean ± SD, statistical analysis using two-tailed paired *t*-test. * *p* < 0.05, ** *p* < 0.01 and **** *p* < 0.0001.

**Figure 8:**
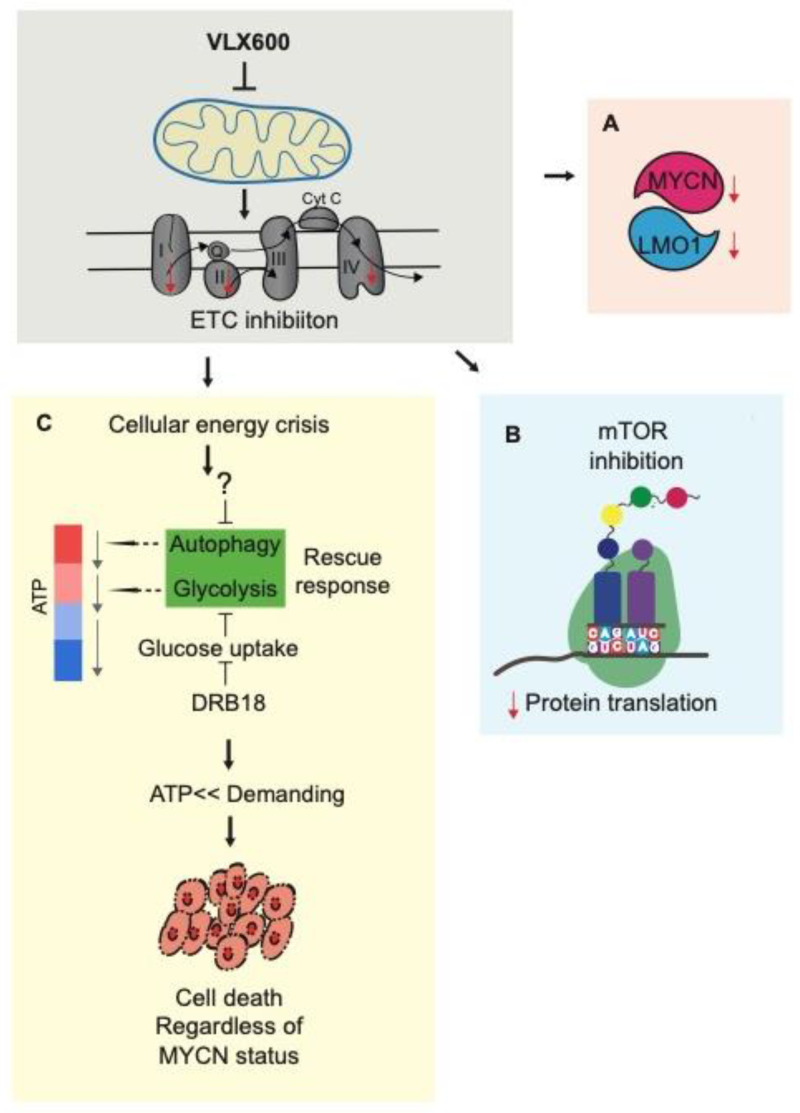
Illustration of the effect of VLX600 in neuroblastoma cells. VLX600 inhibits mitochondrial electron transport chains (ETC) and causes mitochondrial dysfunction and: (**A**) Leads to a reduction in MYCN and LMO1. (**B**) Inhibits mTOR to limit its stimulatory functions on anabolism and protein translation. (**C**) Decreases ATP level mainly produced by mitochondria. Persistent mitochondrial dysfunction by VLX600 eventually results in energy catastrophe which actives autophagy, the protective response to VLX600 in human colon cancer cells but not in neuroblastoma cells, suggesting that neuroblastoma cells have limited tolerance to greater energy expenditure. A further blockage on glucose uptake by DRB18, a potent pan-class I GLUT inhibitor in vitro and in vivo in cancer cells, synergizes the effect of VLX600 in neuroblastoma cells. Since VLX600 has already been investigated in a clinical phase I trial, a therapeutic approach including VLX600 could be an innovative strategy for treating children’s neuroblastoma.

Mitochondrial inhibition leads to an energy crisis due to decreased ATP production. However, autophagy, the protective response to VLX600, was not properly induced in neuroblastoma cells, suggesting that neuroblastoma cells have limited tolerance to greater energy expenditure. Furthermore, VLX600 upregulated glycolysis in neuroblastoma cells (Figure 2G,H), suggesting that blocking glucose uptake may improve the VLX600 effect in neuroblastoma cells. DRB18 is a potent pan-class I GLUT inhibitor in vitro and in vivo in cancer cells [44]. We chose concentrations that have minimal cytotoxicity on their own (Figure 7A,D) and co-treated DRB18 with indicated concentrations of VLX600. A synergistic effect was observed in both IMR-32 and Sk-N-BE(2) cells (Figure 7B,C,E,F), but we did not notice an obvious cell death in hTERT-immortalized retinal pigment epithelial cells (hRPE-1) (Figure 7G,H). Glucose uptake inhibition by DRB18 leads to a reduction of the intracellular glucose amount [44]. Indeed, we started to observe a decrease in the rate of glycolysis in IMR-32 and Sk-N-BE(2) cells exposed to VLX600 after 2 h (Figure 7I,J), which could partially explain the mechanism of the synergistic effect on VLX600 and DRB18 co-treatment (Figure 8).

## 4. Discussion

Neuroblastoma is the most common solid tumor in children featuring the amplification of the *MYCN* protooncogene, a high-risk aggressive clinical marker correlated with treatment failure. Up until today, an indirect inhibition of MYCN expression by targeting upstream or downstream components of the MYCN pathway is still considered an alternative approach.

Previous studies have shown that iron chelators such as Dp44mT, thiosemicarbazone and deferasirox could cause severe mitochondrial dysfunction [45,46,47]. Moreover, thiosemicarbazone has been shown to induce cell death regardless of MYCN expression level [48]. Here, in this paper, we for the first time show that the iron chelator VLX600 inhibits mitochondrial respiration, decreases mTOR activity, reduces the expression of MYCN/LMO1 and induces an efficient cell death regardless of MYCN statues in neuroblastoma cells. Compared to human colon tumor cells, neuroblastoma cells exhibited a significant vulnerability to VLX600 (IC_50_ ~ 6.5 µM vs. IC_50_ ~ 200–400 nM). It is worth pointing out that, even in Sk-N-BE(2) which was taken from the child after repeated courses of chemotherapy and radiotherapy, thus already having a relatively high tolerance to other sources of chemotherapies (e.g., etoposide IC_50_ in Sk-N-BE(2) cells is ~1000 nM but ~152 nM in IMR-32, Supplementary Figure S2), VLX600 still shows a great cytotoxic effect (IC_50_:326 ± 37 nM).

Many studies have observed that, compared to 2D monolayer conditions, cancer cells are more resistant under 3D spheroids conditions, such as doxorubicin, and IC_50_ under 3D condition can be 50-fold that under a 2D condition [49,50,51]. It is worth noting that, in our in vitro study, VLX600 has a very similar IC_50_ under both 2D and 3D conditions. At the same time, in a completed clinical investigation, VLX600 could reach up to 200ng/mL (~666 nM) in plasma even after 10 h injection at a 60mg dosage [52], indicating that VLX600 may achieve a desired clinical efficacy on neuroblastoma.

Inhibition of OXPHOS shifts the mitochondrial metabolism to glycolysis in order to keep the ATP level and maintain essential cellular functions [30]. Consistent with our previous finding in colon cancer cells [29], we also noticed an upregulation of glycolysis in neuroblastoma cells exposed to VLX600. Thus, a further inhibition of glycolysis may generate a greater energy catastrophe and stimulate cell death. To further explore this possibility, we combined VLX600 with DRB18, a potent pan-class I GLUT inhibitor and a synergistic effect was observed (Figure 7). Together with our finding that a protective autophagy response to VLX600 was not sufficiently induced in neuroblastoma cells, targeting pathways or cellular activities related to energy supply/metabolism in neuroblastoma cells may help to find better combinations with VLX600.

Our study also observed several interesting phenomena. (1) Studies have shown that inhibiting mTOR signaling could deregulate *MYCN*-driven proliferation [14,18,19]. However, in this study, dual pan-class I PI3K and mTOR kinase inhibitor BEZ-235 [53] could deregulate mTOR activity but did not decrease the expression level of MYCN, indicating that regulating MYCN through mTOR may as well require another co-factor, such as mitochondrial inhibition. (2) Autophagy consists of steps of initiation/nucleation, elongation, maturation, and finally, fusion and degradation [54]. In our previous study, autophagy was induced and shown to be a protective response to VLX600 in colon cancer cells [33,42]. However, this protective response could not be detected in neuroblastoma cells. The underlying mechanism remains unclear, but according to our recent data (unpublished), insufficient autophagy could be due to an unsuccessful initiation/nucleation in neuroblastoma cells. These phenomena deserve further investigation, allowing us to discover new targets for neuroblastoma therapy.

## 5. Conclusions

MYC amplification plays a vital role in chemoresistance [55]. Here, our results suggest that mitochondria inhibitor VLX600 promotes sensitization of neuroblastoma cells in nutrition-restricted conditions (spheroids condition, insufficient autophagy and glucose deprivation) regardless of MYCN expression. Together with other findings showing that iron chelators could induce cell death regardless of MYCN status [48] and the fact that MYCN/C-MYC is still unable to be targeted, MYCN is an important clinical marker, but may not be a necessary direct target for the treatment of neuroblastoma.

## Data Availability

The data presented in this study are available in this article (and supplementary material).

## Supplementary Materials

The following supporting information can be downloaded at: https://www.mdpi.com/article/10.3390/cancers14133225/s1, Figure S1: Sk-N-BE(2) cells cannot form neat spheroids; Figure S2: Combination effect of VLX600 and Etoposide on both IMR-32 and Sk-N-BE(2) cells; Figure S3: Uncropped Western Blot images; Data S1: List of changes of mitochondrial genes.
