# Supplementary material for: Iron Chelator VLX600 Inhibits Mitochondrial Respiration and Promotes Sensitization of Neuroblastoma Cells in Nutrition-Restricted Conditions
Source: Cancers (Basel). 2022 Jun 30;14(13):3225. doi: 10.3390/cancers14133225 (PMC9264775; doi:10.3390/cancers14133225)
Supplement: Supplementary file 1 [file cancers-14-03225-s001.zip › Supplementary Data. List of changes of mitochondrial genes.pdf]

	baseMean	log2FoldChar	lfcSE	stat	pvalue	padj
NEU4	116	1.66	0.8176	2.03	0.0426	0.135
ALDH1L2	1530	1.61	0.6371	2.53	0.0115	0.0541
MTHFD2	9990	1.31	0.2411	5.42	5.92e-08	5.42e-06
DMGDH	58.9	1.3	0.9127	1.42	0.155	0.321
PYCR1	6570	1.27	0.2059	6.18	6.55e-10	1.28e-07
PMAIP1	1560	1.21	0.3107	3.88	0.000104	0.00165
UCP2	358	1.17	0.2248	5.2	1.97e-07	1.32e-05
CYP27B1	126	1.17	0.4152	2.81	0.005	0.03
TOP1MT	942	1.15	0.4071	2.83	0.00464	0.0283
MPST	2250	1.06	0.1667	6.34	2.37e-10	5.28e-08
TRAP1	8510	1.04	0.1984	5.27	1.4e-07	1,00E-05
PRDX2	8370	1.03	0.1857	5.53	3.18e-08	3.29e-06
NDUFAF2	951	1.03	0.2187	4.69	2.74e-06	0.000106
ADCK3	2850	1.01	0.2066	4.9	9.51e-07	4.64e-05
NDUFA6	2530	0.956	0.2065	4.63	3.65e-06	0.00013
DCXR	1720	0.951	0.1947	4.88	1.04e-06	4.95e-05
MRPL48	1330	0.945	0.1972	4.79	1.63e-06	7.1e-05
DUSP26	1360	0.944	0.4834	1.95	0.0507	0.153
TRMT1	2230	0.941	0.2129	4.42	1,00E-05	0.000287
PAICS	12300	0.919	0.2229	4.12	3.75e-05	0.00076
SLC16A1	10600	0.879	0.2431	3.61	0.000302	0.00372
MRPL33	2830	0.879	0.4789	1.83	0.0666	0.183
PRDX4	3660	0.877	0.2103	4.17	3.01e-05	0.000641
TIMM44	2330	0.873	0.166	5.26	1.44e-07	1.02e-05
AKAP1	4620	0.864	0.2265	3.81	0.000137	0.00204
MRPL45	1590	0.857	0.25	3.43	0.000606	0.00629
CECR5	2210	0.857	0.2958	2.9	0.00376	0.0242
NDUFAF4	1110	0.855	0.1642	5.21	1.91e-07	1.28e-05
SFXN4	1230	0.853	0.2473	3.45	0.000561	0.00593
C12orf65	898	0.852	0.2785	3.06	0.00223	0.0167
RPS15A	23300	0.851	0.1624	5.24	1.62e-07	1.12e-05
TIMM9	796	0.839	0.1417	5.92	3.24e-09	4.97e-07
QTRT1	1520	0.835	0.1959	4.26	2.04e-05	0.000479
PCK2	1840	0.828	0.3211	2.58	0.00995	0.0489
ALDH18A1	6620	0.825	0.201	4.1	4.1e-05	0.000814
ENDOG	1180	0.822	0.2007	4.09	4.23e-05	0.000836
THNSL1	620	0.821	0.1818	4.51	6.41e-06	0.000202
UNG	3810	0.811	0.2089	3.88	0.000103	0.00163
HSPA9	21700	0.811	0.2119	3.83	0.000129	0.00195
ECSIT	1870	0.808	0.2007	4.03	5.65e-05	0.00103
FASN	14800	0.808	0.2028	3.98	6.78e-05	0.00119
ARG2	821	0.804	0.4233	1.9	0.0576	0.167
NOA1	1660	0.799	0.1687	4.74	2.16e-06	8.77e-05
CKMT1B	293	0.794	0.5474	1.45	0.147	0.31
MRPL1	890	0.789	0.1643	4.8	1.55e-06	6.84e-05

GPT2	2930	0.788	0.2234	3.53	0.000418	0.00478
TSFM	2220	0.786	0.3219	2.44	0.0147	0.0643
COA7	1400	0.777	0.2061	3.77	0.000164	0.00234
BID	1960	0.772	0.1546	4.99	5.98e-07	3.18e-05
DNA2	3060	0.768	0.2243	3.42	0.000623	0.0064
CHCHD10	611	0.761	0.4927	1.54	0.123	0.274
GLDC	1000	0.76	0.6757	1.12	0.261	0.448
GUF1	3090	0.759	0.1657	4.58	4.65e-06	0.000155
ACSL1	3060	0.756	0.2776	2.72	0.00647	0.0359
LIAS	534	0.754	0.1565	4.82	1.42e-06	6.41e-05
SMDT1	543	0.746	0.2894	2.58	0.00993	0.0489
MTHFD1	5480	0.734	0.1873	3.92	8.89e-05	0.00146
COX5A	2680	0.732	0.1858	3.94	8.11e-05	0.00137
ALDH7A1	2530	0.723	0.1743	4.15	3.32e-05	0.000693
GCAT	669	0.72	0.2664	2.7	0.00692	0.0378
LDHB	34400	0.719	0.1733	4.15	3.36e-05	7,00E-04
SLC25A37	2130	0.719	0.2281	3.15	0.00161	0.0131
MRPL15	1940	0.712	0.1811	3.93	8.36e-05	0.0014
PIF1	935	0.712	0.2702	2.63	0.00845	0.0436
SLC25A33	781	0.702	0.2399	2.92	0.00345	0.0228
NARS2	1990	0.701	0.1609	4.36	1.31e-05	0.000345
ABCB9	706	0.699	0.3801	1.84	0.0658	0.182
CKMT1A	260	0.697	0.4749	1.47	0.142	0.303
SLC25A19	657	0.696	0.2267	3.07	0.00214	0.0161
RPUSD4	1570	0.675	0.2108	3.2	0.00137	0.0116
MRPL11	1900	0.674	0.1985	3.39	0.000687	0.0069
MTPAP	2740	0.673	0.1451	4.64	3.56e-06	0.000128
NLN	3600	0.671	0.1428	4.7	2.66e-06	0.000103
MRPL54	810	0.669	0.1846	3.62	0.000289	0.00362
C6orf136	0.821	0.669	1.7278	0.387	0.699	0.82
TIMM13	2930	0.657	0.1848	3.55	0.000379	0.00444
HSPE1	1630	0.657	0.2553	2.57	0.0101	0.0493
CCDC58	718	0.653	0.2183	2.99	0.00276	0.0194
MRPL12	1960	0.651	0.2902	2.24	0.0248	0.0932
MRPS27	4020	0.649	0.1462	4.44	9.1e-06	0.000268
AADAT	930	0.637	0.1583	4.02	5.72e-05	0.00104
TARS	9710	0.637	0.2075	3.07	0.00216	0.0162
FLAD1	3810	0.633	0.2116	2.99	0.00277	0.0195
ACACA	11000	0.632	0.1851	3.41	0.000643	0.00656
LIPT2	89.6	0.631	0.29	2.17	0.0297	0.106
COA4	1880	0.63	0.1785	3.53	0.000417	0.00477
MTFR1	1120	0.626	0.211	2.96	0.00304	0.0208
NTHL1	1200	0.625	0.1546	4.04	5.24e-05	0.000974
RPL10A	21700	0.623	0.154	4.05	5.22e-05	0.000972
SECISBP2	3170	0.622	0.1547	4.02	5.81e-05	0.00105
ATP5G1	2620	0.622	0.2436	2.55	0.0107	0.0513

NDUFS7	1790	0.621	0.161	3.86	0.000114	0.00177
AGPAT5	6020	0.621	0.1736	3.58	0.000345	0.00412
LRPPRC	17700	0.619	0.1819	3.4	0.000673	0.0068
SFXN2	760	0.617	0.2965	2.08	0.0375	0.124
C15orf61	366	0.613	0.1417	4.33	1.51e-05	0.000383
DNAJA3	2770	0.609	0.172	3.54	0.000399	0.0046
MPC1	832	0.605	0.2661	2.27	0.023	0.0886
NDUFA7	1180	0.6	0.1632	3.67	0.000238	0.00309
SLC25A53	228	0.6	0.2181	2.75	0.00589	0.0336
HSPD1	32400	0.6	0.2353	2.55	0.0108	0.0519
TRNT1	1330	0.599	0.1879	3.19	0.00144	0.0121
FASTKD3	712	0.597	0.1374	4.34	1.42e-05	0.000366
NT5M	524	0.597	0.2822	2.12	0.0343	0.116
RECQL4	2610	0.596	0.1974	3.02	0.00253	0.0183
HADH	1260	0.595	0.1955	3.04	0.00235	0.0174
REXO2	1270	0.595	0.3502	1.7	0.0894	0.223
MRPL38	2220	0.594	0.1515	3.92	8.92e-05	0.00146
NDUFV3	1550	0.592	0.1452	4.08	4.59e-05	0.000885
ATIC	1590	0.588	0.1445	4.07	4.69e-05	0.000899
MRPL36	770	0.583	0.176	3.31	0.000934	0.00876
PTGES2	2050	0.58	0.1672	3.47	0.000527	0.00566
FKBP10	7550	0.578	0.2703	2.14	0.0326	0.112
MCAT	708	0.576	0.1476	3.9	9.44e-05	0.00153
GADD45GIP1	1710	0.572	0.2296	2.49	0.0128	0.0585
TRMT11	1520	0.571	0.1907	3	0.00274	0.0193
FXN	537	0.571	0.212	2.69	0.00705	0.0382
ATP5O	4510	0.569	0.1329	4.28	1.84e-05	0.00044
POLRMT	2940	0.566	0.1796	3.15	0.00163	0.0133
ACLY	22100	0.566	0.1925	2.94	0.00326	0.0219
LYRM4	1270	0.562	0.1472	3.82	0.000132	0.00198
HK2	4320	0.562	0.3312	1.7	0.0899	0.224
RPL35A	17100	0.561	0.1539	3.65	0.000265	0.00337
CISD1	1260	0.561	0.1865	3.01	0.00265	0.0189
SLC37A4	1360	0.561	0.206	2.73	0.00643	0.0358
MRPL37	2610	0.56	0.2503	2.24	0.0252	0.0943
SUGCT	208	0.559	0.2889	1.94	0.0529	0.158
PTCD1	15.1	0.558	0.2181	2.56	0.0106	0.051
MAOA	7860	0.557	0.6309	0.883	0.377	0.564
PUS1	1010	0.555	0.2171	2.56	0.0106	0.0511
EPHX2	472	0.555	0.5898	0.94	0.347	0.535
PXMP2	1090	0.55	0.2098	2.62	0.0087	0.0445
MRPL18	1610	0.549	0.1997	2.75	0.00603	0.0341
NIPSNAP1	6310	0.548	0.1628	3.36	0.000769	0.0075
ETFA	3330	0.547	0.1736	3.15	0.00165	0.0133
CCBL2	1060	0.546	0.2623	2.08	0.0372	0.123
ACAT1	2440	0.543	0.3181	1.71	0.088	0.22

MRPL4	1410	0.54	0.2162	2.5	0.0126	0.0579
NDUFB8	3320	0.538	0.1519	3.54	0.000394	0.00456
ECI1	1180	0.538	0.1863	2.89	0.0039	0.0249
AARS2	4600	0.536	0.2732	1.96	0.0496	0.151
MTFP1	771	0.534	0.2963	1.8	0.0716	0.192
ALDH1B1	2360	0.533	0.1918	2.78	0.00546	0.0318
GRPEL1	1810	0.531	0.181	2.94	0.00332	0.0222
MRRF	1250	0.528	0.124	4.25	2.09e-05	0.000487
SUPV3L1	1730	0.528	0.126	4.19	2.73e-05	0.000597
CHDH	148	0.527	0.6859	0.768	0.442	0.623
MARS2	935	0.525	0.2383	2.2	0.0275	0.1
TYSND1	959	0.523	0.1952	2.68	0.00736	0.0395
DDX28	629	0.521	0.1773	2.94	0.00326	0.0219
TKT	11500	0.52	0.2198	2.36	0.0181	0.0747
GCDH	756	0.519	0.2017	2.57	0.0101	0.0494
NDUFS6	1390	0.518	0.1926	2.69	0.00717	0.0387
DLAT	3020	0.518	0.2226	2.33	0.02	0.0803
EARS2	2620	0.515	0.0966	5.33	9.59e-08	7.48e-06
MRPS30	1970	0.515	0.1275	4.04	5.37e-05	0.000993
ACADM	2260	0.512	0.2231	2.29	0.0219	0.0855
MRPL21	1680	0.51	0.1716	2.97	0.00296	0.0204
COQ3	467	0.507	0.1785	2.84	0.00453	0.0278
TIMM50	3180	0.505	0.1881	2.68	0.00729	0.0392
MRPS2	1870	0.505	0.2208	2.29	0.0223	0.0865
MRPL14	1540	0.499	0.1453	3.44	0.000588	0.00614
PRDX3	5510	0.497	0.2047	2.43	0.0152	0.0661
GOT2	5460	0.496	0.1526	3.25	0.00115	0.0102
CRY1	1460	0.496	0.1756	2.83	0.00472	0.0286
CCT7	15500	0.495	0.1452	3.41	0.000659	0.00669
RMND1	1220	0.489	0.2053	2.38	0.0171	0.0719
COX11	2790	0.487	0.1393	3.5	0.000473	0.00523
NDUFA8	1100	0.487	0.1699	2.86	0.00418	0.0262
NDUFC2	890	0.487	0.176	2.77	0.00566	0.0326
GARS	8530	0.487	0.2282	2.14	0.0327	0.113
CYCS	8780	0.486	0.1792	2.71	0.00673	0.037
DCAKD	2070	0.485	0.2059	2.36	0.0184	0.0758
FHIT	129	0.484	0.3495	1.38	0.166	0.336
MRPS34	2850	0.482	0.1596	3.02	0.00253	0.0182
BOLA1	386	0.48	0.2268	2.12	0.0343	0.116
LYRM1	789	0.479	0.1792	2.67	0.00755	0.0403
MRPL30	3990	0.478	0.1256	3.81	0.000141	0.00209
MRPL22	672	0.478	0.1727	2.77	0.0056	0.0324
METAP1D	300	0.478	0.2219	2.16	0.0311	0.109
RPUSD3	790	0.477	0.2069	2.31	0.0211	0.0832
CLPP	1430	0.475	0.1354	3.51	0.000448	0.00504
RPL34	14400	0.475	0.1588	2.99	0.00278	0.0195

SARS2	861	0.473	0.1528	3.1	0.00194	0.0151
GPAM	4890	0.472	0.2523	1.87	0.0615	0.174
ATP5F1	7500	0.47	0.2194	2.14	0.0322	0.111
TMEM126B	1180	0.469	0.1853	2.53	0.0113	0.0537
ACSF3	674	0.469	0.3172	1.48	0.139	0.299
RTN4IP1	545	0.468	0.1438	3.26	0.00113	0.0101
MRPL3	5340	0.468	0.1595	2.93	0.00337	0.0224
C14orf159	1160	0.465	0.2157	2.16	0.0311	0.109
TMEM126A	628	0.464	0.2091	2.22	0.0265	0.0975
LYPLA1	3850	0.463	0.1685	2.75	0.00598	0.0339
MRPL42	3790	0.462	0.1574	2.93	0.00334	0.0223
MRPL32	1820	0.461	0.1608	2.87	0.00412	0.0259
PAM16	7.95	0.461	0.3337	1.38	0.167	0.337
MTHFD2L	424	0.458	0.1711	2.67	0.00748	0.04
IDH2	5100	0.455	0.2015	2.26	0.0238	0.0907
RMDN3	1230	0.454	0.1409	3.22	0.00126	0.0109
NDUFV1	5510	0.453	0.1281	3.54	0.000405	0.00467
COX7B	3470	0.451	0.1888	2.39	0.0169	0.0713
TOMM20	21800	0.451	0.1933	2.33	0.0196	0.0793
MACROD1	1000	0.45	0.2971	1.51	0.13	0.286
DECR1	1050	0.448	0.2941	1.52	0.128	0.282
MRPS7	3260	0.447	0.2109	2.12	0.0343	0.116
DIABLO	5000	0.446	0.3688	1.21	0.226	0.41
UQCRC2	6000	0.445	0.1362	3.27	0.00109	0.00984
QRSL1	1320	0.444	0.1295	3.42	0.000616	0.00636
SOD1	6600	0.444	0.133	3.34	0.000836	0.00799
PXMP4	1070	0.444	0.3458	1.28	0.199	0.379
PTCD3	6330	0.443	0.1179	3.76	0.00017	0.00239
GRSF1	5970	0.443	0.1615	2.74	0.00613	0.0345
NDUFA11	2680	0.442	0.1439	3.07	0.00212	0.016
ICT1	1330	0.442	0.1973	2.24	0.0251	0.094
DHODH	650	0.441	0.1745	2.53	0.0114	0.054
ATP5L	3280	0.44	0.2285	1.93	0.0542	0.16
PSTK	174	0.439	0.238	1.84	0.0651	0.18
MRPS9	1210	0.438	0.1579	2.77	0.00556	0.0322
CLPB	5340	0.438	0.1997	2.19	0.0283	0.102
CHCHD6	954	0.436	0.2111	2.06	0.0391	0.127
TTC19	2680	0.435	0.1764	2.46	0.0138	0.0616
CYC1	3760	0.432	0.1707	2.53	0.0114	0.0538
RPIA	685	0.43	0.1437	3	0.00274	0.0194
SHMT1	885	0.43	0.2389	1.8	0.0719	0.192
MRPL40	1080	0.429	0.1827	2.35	0.0189	0.0771
C1QBP	6470	0.428	0.2303	1.86	0.0628	0.176
ACAA2	3400	0.427	0.1927	2.22	0.0266	0.0978
MRPS25	3660	0.425	0.2073	2.05	0.0405	0.131
MTO1	1060	0.424	0.167	2.54	0.0112	0.0532

IBA57	673	0.424	0.1865	2.27	0.023	0.0887
SLC16A7	1720	0.424	0.5817	0.728	0.466	0.644
UQCR10	2220	0.421	0.1649	2.56	0.0106	0.0511
DTYMK	2090	0.419	0.2186	1.91	0.0555	0.163
SIRT4	85.8	0.418	0.2886	1.45	0.148	0.312
TFAM	3660	0.416	0.1557	2.67	0.00763	0.0406
VDAC2	5530	0.416	0.1595	2.61	0.00908	0.0458
NUDT9	742	0.415	0.226	1.84	0.0661	0.182
PNPT1	3440	0.414	0.1753	2.36	0.0181	0.0749
RNMTL1	725	0.41	0.1758	2.33	0.0196	0.0791
PNPO	1630	0.41	0.254	1.61	0.107	0.251
PTCD2	487	0.406	0.1157	3.51	0.000453	0.00508
MRPL39	1140	0.405	0.1352	2.99	0.00275	0.0194
NDUFAF5	909	0.404	0.1239	3.26	0.00111	0.00999
SLC25A39	5120	0.404	0.2371	1.7	0.0884	0.221
LETM2	264	0.397	0.198	2	0.0452	0.141
GCSH	810	0.394	0.2283	1.73	0.0842	0.214
TACO1	1200	0.393	0.1705	2.3	0.0212	0.0834
AIFM1	1260	0.392	0.2273	1.73	0.0845	0.214
MTCH2	4640	0.391	0.1285	3.05	0.00231	0.0172
HMBS	510	0.39	0.3116	1.25	0.211	0.392
ACSS1	201	0.39	0.9181	0.425	0.671	0.801
NT5DC2	6220	0.388	0.1899	2.04	0.0411	0.132
TARS2	2070	0.388	0.1991	1.95	0.0515	0.154
DHTKD1	3740	0.387	0.132	2.93	0.00339	0.0225
ME2	3350	0.387	0.154	2.51	0.012	0.0559
MRPL57	1320	0.386	0.1232	3.14	0.00171	0.0137
GSR	3890	0.384	0.1555	2.47	0.0136	0.061
MGME1	1440	0.384	0.2013	1.91	0.0566	0.165
ATP5C1	6000	0.383	0.1546	2.48	0.0133	0.06
COX8C	6.82	0.382	0.6125	0.624	0.533	0.7
MRPS17	738	0.381	0.194	1.96	0.0496	0.151
COX17	925	0.38	0.324	1.17	0.241	0.427
NDUFB11	3320	0.377	0.1325	2.85	0.00439	0.0272
LETMD1	2130	0.377	0.1438	2.62	0.00873	0.0447
PTRH2	1800	0.375	0.1968	1.91	0.0567	0.165
DUS2	677	0.372	0.1494	2.49	0.0128	0.0585
TMEM70	927	0.371	0.1009	3.68	0.000232	0.00303
EMC2	1070	0.369	0.1643	2.24	0.0248	0.0932
WARS2	607	0.368	0.1545	2.38	0.0172	0.0722
ATP5B	30100	0.368	0.1628	2.26	0.0238	0.0907
OSGEPL1	916	0.368	0.1737	2.12	0.0343	0.116
PACSIN2	2350	0.367	0.1543	2.38	0.0175	0.0731
HINT1	7950	0.367	0.1625	2.26	0.0238	0.0907
MRPL23	1490	0.364	0.1797	2.02	0.043	0.136
HIGD2A	1420	0.363	0.203	1.79	0.0738	0.196

NME4	3460	0.362	0.1866	1.94	0.0522	0.156
GLOD4	2700	0.359	0.1908	1.88	0.0602	0.171
SND1	11400	0.357	0.1536	2.32	0.0203	0.0811
NUDT5	2340	0.357	0.1689	2.11	0.0347	0.117
NDUFA5	2990	0.354	0.1402	2.53	0.0115	0.0542
ATP5G2	6350	0.354	0.1605	2.2	0.0275	0.1
RPS14	26900	0.354	0.2101	1.68	0.0924	0.228
MRPL2	584	0.353	0.1811	1.95	0.0511	0.154
TMEM177	604	0.352	0.1548	2.27	0.0231	0.0889
RBFA	1440	0.352	0.1924	1.83	0.067	0.184
PCBD2	452	0.35	0.2277	1.54	0.124	0.277
MCCC2	1940	0.347	0.1599	2.17	0.03	0.106
XPNPEP3	1590	0.347	0.1806	1.92	0.0547	0.161
MRPS18C	723	0.345	0.1498	2.3	0.0214	0.084
MRPS35	2590	0.344	0.1487	2.31	0.0207	0.0821
VDAC3	3910	0.342	0.1309	2.61	0.00894	0.0453
MRPS15	1620	0.342	0.2744	1.25	0.212	0.394
NFU1	736	0.34	0.1208	2.81	0.00493	0.0297
COMT	1470	0.338	0.2259	1.5	0.134	0.291
PPA2	1630	0.336	0.1985	1.69	0.0904	0.225
OGDHL	2520	0.336	0.3886	0.865	0.387	0.572
PKLR	2.13	0.336	0.7261	0.462	0.644	0.783
PPIF	2300	0.335	0.2356	1.42	0.155	0.322
UQCRFS1	2610	0.334	0.1721	1.94	0.0521	0.156
TMEM11	664	0.332	0.1392	2.39	0.017	0.0716
FOXRED1	1250	0.332	0.1916	1.73	0.0832	0.212
GAPDH	117000	0.332	0.2063	1.61	0.107	0.251
WDR81	1540	0.331	0.2108	1.57	0.116	0.265
STOML2	4260	0.33	0.1622	2.04	0.0417	0.133
NDUFA4	6290	0.33	0.1777	1.86	0.0636	0.178
NAGS	84.2	0.326	0.494	0.659	0.51	0.68
LACTB2	385	0.325	0.2388	1.36	0.174	0.346
RHOT1	2820	0.324	0.1771	1.83	0.0671	0.184
SLC25A23	1080	0.324	0.2101	1.54	0.123	0.274
SCO2	484	0.322	0.1967	1.64	0.101	0.242
NDUFA10	2910	0.321	0.1371	2.34	0.0191	0.0779
FAM210B	1640	0.321	0.3101	1.04	0.3	0.489
ABCB7	1570	0.318	0.1153	2.76	0.00586	0.0335
CS	6900	0.318	0.1415	2.24	0.0248	0.0933
AK3	2540	0.318	0.1827	1.74	0.0823	0.21
GSTZ1	746	0.318	0.231	1.38	0.169	0.339
BCL2	3050	0.317	0.4554	0.696	0.486	0.66
NDUFAB1	2660	0.315	0.1345	2.35	0.019	0.0775
KIAA0100	12800	0.315	0.1585	1.98	0.0472	0.146
ECHDC1	2030	0.315	0.1779	1.77	0.0771	0.201
CISD2	1670	0.314	0.1657	1.9	0.058	0.167

COA3	1450	0.314	0.1905	1.65	0.0995	0.24
ABHD10	1770	0.313	0.1495	2.09	0.0364	0.121
TOMM5	3480	0.313	0.2068	1.52	0.13	0.285
NDUFAF1	538	0.31	0.1369	2.27	0.0233	0.0896
UQCR11	2430	0.31	0.1437	2.15	0.0313	0.109
SDR39U1	868	0.309	0.1176	2.63	0.00861	0.0442
FUNDC2	2160	0.309	0.1441	2.14	0.0322	0.111
ERAL1	1360	0.309	0.1624	1.91	0.0568	0.165
MRPL9	2340	0.309	0.1683	1.84	0.0663	0.182
CDC25C	682	0.308	0.257	1.2	0.231	0.415
DNAJC19	1150	0.306	0.1315	2.33	0.0198	0.0798
C19orf52	624	0.306	0.1517	2.02	0.0435	0.138
STARD7	9800	0.305	0.1147	2.66	0.00791	0.0416
ABCF2	4260	0.305	0.1859	1.64	0.101	0.242
CLPX	2270	0.304	0.1107	2.75	0.006	0.034
NDUFS3	1890	0.303	0.1232	2.46	0.014	0.0621
LONP1	6320	0.303	0.1731	1.75	0.0798	0.206
CHCHD4	589	0.302	0.1897	1.59	0.111	0.257
MRPL50	1190	0.301	0.134	2.25	0.0247	0.0929
NDUFB4	2560	0.301	0.1801	1.67	0.095	0.232
FDPS	6850	0.301	0.199	1.51	0.131	0.286
MRPS14	1540	0.299	0.1536	1.94	0.0519	0.155
SUCLG1	2470	0.298	0.135	2.2	0.0275	0.1
SAMM50	2200	0.298	0.1499	1.99	0.0466	0.144
TIMM8B	1330	0.298	0.2151	1.38	0.166	0.336
ATP5S	463	0.296	0.1188	2.49	0.0128	0.0584
ATP5A1	20800	0.296	0.1296	2.29	0.0223	0.0865
DHCR24	10500	0.296	0.2271	1.3	0.192	0.369
MRPS31	1010	0.295	0.1154	2.56	0.0105	0.0507
TOMM22	3970	0.295	0.1677	1.76	0.0782	0.203
BCKDHA	1240	0.295	0.1704	1.73	0.0836	0.213
MCEE	202	0.294	0.1305	2.25	0.0242	0.0916
ATP5H	4270	0.294	0.1621	1.81	0.0696	0.188
MRPL44	1320	0.293	0.1108	2.65	0.00812	0.0424
FDX1L	486	0.291	0.1784	1.63	0.103	0.245
MDH2	6990	0.291	0.1903	1.53	0.126	0.279
NDUFS8	1950	0.29	0.1272	2.28	0.0225	0.0873
COQ5	1400	0.29	0.1687	1.72	0.0853	0.216
TMEM205	1420	0.29	0.2629	1.1	0.27	0.458
SLC25A3	15900	0.289	0.1639	1.77	0.0773	0.202
GLUD1	6230	0.289	0.168	1.72	0.0859	0.217
NDUFA9	3160	0.287	0.1663	1.73	0.0845	0.214
COX6C	3890	0.285	0.1748	1.63	0.103	0.245
PHB	8260	0.284	0.2494	1.14	0.254	0.441
NDUFA12	1790	0.282	0.1472	1.92	0.055	0.162
IVD	1820	0.282	0.149	1.89	0.0584	0.168

FAM136A	1820	0.278	0.1472	1.89	0.0594	0.17
BCL2L13	3060	0.278	0.1545	1.8	0.0723	0.193
HADHA	10200	0.277	0.1546	1.79	0.0736	0.195
TOMM40	3010	0.276	0.1981	1.39	0.163	0.332
NUDT2	506	0.276	0.201	1.37	0.17	0.34
RAB24	999	0.276	0.222	1.24	0.214	0.396
CYB5B	4400	0.275	0.2378	1.16	0.247	0.433
PHB2	11600	0.274	0.1615	1.7	0.0895	0.223
IMMP2L	388	0.274	0.233	1.18	0.24	0.425
PRDX6	6690	0.274	0.2417	1.13	0.257	0.444
METTL5	1150	0.272	0.1735	1.57	0.117	0.266
PPWD1	1440	0.271	0.0993	2.73	0.00642	0.0358
GRPEL2	2990	0.271	0.1667	1.63	0.104	0.246
AK2	5290	0.271	0.2366	1.15	0.252	0.438
YARS2	1230	0.27	0.1472	1.83	0.0671	0.184
BCKDHB	1170	0.27	0.17	1.59	0.113	0.26
OCIAD1	5020	0.269	0.1117	2.41	0.0161	0.0689
MLH1	2820	0.267	0.1558	1.71	0.0871	0.219
NDUFS4	1610	0.265	0.1301	2.04	0.0416	0.133
OMA1	605	0.265	0.2527	1.05	0.295	0.484
ALDH2	3010	0.264	0.2308	1.14	0.253	0.439
MIEF1	3720	0.263	0.1396	1.89	0.0593	0.17
GTPBP3	1140	0.263	0.1956	1.34	0.179	0.353
MTIF2	2360	0.259	0.1909	1.35	0.175	0.348
COQ7	1070	0.258	0.1249	2.06	0.0391	0.128
SLC25A25	1860	0.258	0.1656	1.56	0.12	0.27
ATP5D	1020	0.257	0.1884	1.37	0.172	0.343
FECH	2120	0.257	0.2076	1.24	0.216	0.398
UQCC2	1590	0.255	0.1221	2.09	0.0367	0.122
COX7C	6930	0.255	0.153	1.67	0.0955	0.233
C17orf89	1350	0.254	0.2053	1.24	0.216	0.399
CHCHD2	11900	0.253	0.1755	1.44	0.149	0.313
OCIAD2	884	0.253	0.2679	0.945	0.345	0.533
TAMM41	363	0.252	0.1694	1.49	0.138	0.296
ATP5G3	4910	0.252	0.1712	1.47	0.141	0.301
PC	1490	0.252	0.2568	0.982	0.326	0.515
MRPS24	3.44	0.252	0.3987	0.632	0.527	0.695
SDHA	3840	0.251	0.1579	1.59	0.112	0.259
UQCRB	4320	0.248	0.1768	1.4	0.161	0.329
PDSS1	697	0.248	0.1883	1.32	0.187	0.363
GPI	11400	0.248	0.1895	1.31	0.191	0.368
MTFMT	635	0.247	0.0939	2.63	0.00845	0.0436
PET100	1080	0.247	0.1281	1.93	0.0534	0.159
ACOT13	695	0.247	0.2121	1.17	0.244	0.43
MRPS6	633	0.247	0.2276	1.08	0.278	0.466
FASTKD1	1730	0.246	0.1424	1.73	0.084	0.214

FAM213A	3670	0.246	0.1754	1.4	0.161	0.329
RSAD1	2010	0.244	0.2121	1.15	0.25	0.437
CMC1	350	0.243	0.2454	0.992	0.321	0.511
NDUFB6	1700	0.241	0.1512	1.6	0.11	0.256
HSCB	268	0.241	0.1598	1.51	0.132	0.289
COX18	926	0.239	0.1286	1.86	0.0636	0.178
NDUFB10	2250	0.239	0.1384	1.73	0.0845	0.214
MRPL46	820	0.239	0.1452	1.65	0.0993	0.239
SLIRP	1910	0.239	0.2038	1.17	0.241	0.426
NT5DC3	1680	0.239	0.3191	0.749	0.454	0.632
PI4KA	6350	0.237	0.1783	1.33	0.184	0.36
MMACHC	547	0.234	0.2667	0.878	0.38	0.566
BCS1L	1240	0.233	0.1634	1.42	0.154	0.321
STAR	85.3	0.233	0.5544	0.42	0.674	0.803
SLC25A17	1500	0.232	0.1641	1.41	0.158	0.325
GLYCTK	464	0.23	0.266	0.866	0.386	0.572
LAP3	2150	0.229	0.1541	1.48	0.138	0.296
FDX1	516	0.228	0.2545	0.895	0.371	0.558
TRIAP1	1140	0.224	0.1841	1.22	0.224	0.407
OXSM	402	0.222	0.1903	1.17	0.243	0.429
ABCB10	1990	0.219	0.143	1.53	0.126	0.279
DUT	4820	0.219	0.143	1.53	0.126	0.279
C5orf63	565	0.219	0.5547	0.395	0.693	0.816
SLC25A11	1620	0.217	0.1796	1.21	0.227	0.411
METTL8	1350	0.214	0.1468	1.46	0.145	0.308
PUSL1	448	0.212	0.2796	0.758	0.448	0.628
GATC	3060	0.211	0.1456	1.45	0.147	0.31
NNT	6130	0.209	0.1194	1.75	0.0799	0.206
DAP3	4500	0.209	0.2243	0.931	0.352	0.539
YBEY	453	0.208	0.1685	1.24	0.217	0.399
IDE	2760	0.207	0.2018	1.03	0.305	0.494
ELAC2	3410	0.205	0.1668	1.23	0.22	0.403
SPRYD4	598	0.203	0.1648	1.23	0.218	0.4
OXNAD1	1050	0.202	0.1561	1.3	0.195	0.373
TEFM	421	0.202	0.1682	1.2	0.229	0.413
MRPL27	1930	0.201	0.1652	1.22	0.223	0.407
COX15	3060	0.199	0.1504	1.32	0.187	0.362
SOD2	1950	0.198	0.2044	0.969	0.333	0.522
C14orf2	2430	0.197	0.1629	1.21	0.227	0.41
UQCRC1	4880	0.197	0.1781	1.11	0.268	0.456
THG1L	855	0.197	0.1828	1.08	0.282	0.47
GBAS	2250	0.196	0.1813	1.08	0.279	0.467
TXN2	1170	0.195	0.1526	1.28	0.2	0.38
SERAC1	592	0.195	0.2464	0.79	0.43	0.613
MTX1	1090	0.193	0.2224	0.869	0.385	0.57
COMTD1	306	0.193	0.3023	0.637	0.524	0.693

OPA1	8300	0.192	0.1235	1.56	0.12	0.27
COX7A2L	3850	0.192	0.1482	1.3	0.195	0.373
NSUN4	1060	0.192	0.2215	0.866	0.387	0.572
SLC25A32	1950	0.189	0.1603	1.18	0.239	0.424
MRPL16	906	0.188	0.1365	1.38	0.168	0.338
PPTC7	728	0.188	0.186	1.01	0.312	0.501
GPX4	4110	0.188	0.1879	1	0.317	0.506
OSBPL1A	2430	0.187	0.1912	0.98	0.327	0.516
TIMM10	917	0.183	0.2005	0.913	0.361	0.548
SFXN1	3950	0.181	0.1601	1.13	0.257	0.444
KARS	7930	0.181	0.1782	1.01	0.31	0.499
TOMM34	1890	0.181	0.2253	0.803	0.422	0.605
GLRX5	1330	0.18	0.1566	1.15	0.252	0.439
ATPAF2	484	0.18	0.1679	1.07	0.283	0.471
NCOA4	6130	0.18	0.1844	0.975	0.329	0.518
BOLA3	602	0.18	0.2273	0.792	0.429	0.612
COQ2	420	0.179	0.1918	0.934	0.35	0.538
ETFDH	613	0.179	0.1933	0.924	0.355	0.542
VDAC1	9200	0.179	0.231	0.773	0.439	0.62
MPV17L	207	0.178	0.8217	0.216	0.829	0.902
CRLS1	1680	0.177	0.1268	1.4	0.163	0.331
ACSM3	12.9	0.176	0.6558	0.269	0.788	0.878
EEFSEC	610	0.174	0.1978	0.881	0.378	0.564
GFER	1020	0.173	0.1223	1.42	0.157	0.324
COX8A	5510	0.171	0.1429	1.2	0.232	0.416
COASY	1670	0.171	0.2226	0.77	0.441	0.622
CCDC127	824	0.169	0.1203	1.4	0.161	0.329
MMADHC	3070	0.169	0.1777	0.952	0.341	0.529
MCCC1	1680	0.168	0.1515	1.11	0.267	0.455
PDHA1	3350	0.165	0.1327	1.25	0.213	0.395
MRPL49	1940	0.164	0.1402	1.17	0.242	0.427
LARS2	2100	0.163	0.1875	0.868	0.386	0.571
DARS2	3540	0.163	0.2372	0.689	0.491	0.664
CYP24A1	3.83	0.162	1.9797	0.082	0.935	0.966
LYRM7	1720	0.161	0.1574	1.02	0.306	0.495
LYPLAL1	817	0.161	0.1904	0.845	0.398	0.582
ACO2	3520	0.16	0.1965	0.815	0.415	0.598
SDHD	1680	0.16	0.2504	0.637	0.524	0.693
NDUFB9	4430	0.159	0.197	0.808	0.419	0.602
IMMT	5600	0.158	0.1282	1.23	0.217	0.4
MRPS5	1920	0.156	0.0945	1.65	0.0997	0.24
C2orf47	687	0.154	0.1341	1.14	0.252	0.439
MRPL10	2330	0.154	0.1777	0.866	0.386	0.572
C2orf69	1230	0.153	0.1214	1.26	0.206	0.387
ATP5J2	1400	0.152	0.1801	0.842	0.4	0.584
PTS	764	0.15	0.2562	0.586	0.558	0.719

GTPBP10	1410	0.149	0.1394	1.07	0.286	0.474
ALDH3A2	2090	0.149	0.1718	0.868	0.386	0.571
PDP2	1150	0.146	0.1666	0.876	0.381	0.567
RCN2	4750	0.145	0.1727	0.839	0.401	0.586
CHCHD3	4520	0.144	0.1914	0.752	0.452	0.631
GNG5	1400	0.142	0.3581	0.396	0.692	0.816
01-Mar	1310	0.142	0.4534	0.314	0.754	0.856
PCCB	2280	0.14	0.1532	0.916	0.36	0.547
PROSC	954	0.139	0.15	0.925	0.355	0.542
SLC25A15	2070	0.139	0.1688	0.823	0.411	0.594
FAM195A	599	0.138	0.2305	0.6	0.549	0.712
QDPR	1160	0.138	0.2776	0.496	0.62	0.766
POLDIP2	3040	0.136	0.1712	0.797	0.425	0.608
MRPS10	1770	0.133	0.1519	0.873	0.383	0.569
MRPS18A	1310	0.132	0.1647	0.804	0.421	0.604
SLC25A45	265	0.132	0.3341	0.394	0.693	0.816
LYRM5	370	0.131	0.1433	0.912	0.362	0.548
GFM2	3190	0.128	0.1356	0.946	0.344	0.532
MRM1	390	0.128	0.2311	0.554	0.579	0.734
ABCA9	30.4	0.128	1.7386	0.0734	0.941	0.97
ALAS1	1450	0.126	0.1705	0.741	0.459	0.637
D2HGDH	646	0.126	0.1971	0.639	0.523	0.692
MRPL13	1260	0.126	0.2227	0.568	0.57	0.728
FBXL4	1340	0.122	0.1421	0.855	0.393	0.577
FASTKD2	3070	0.121	0.1378	0.879	0.38	0.566
MFF	3140	0.12	0.0979	1.23	0.22	0.403
DHX30	5890	0.12	0.1646	0.727	0.467	0.644
ARL2	700	0.12	0.1993	0.604	0.546	0.71
ALDH4A1	822	0.12	0.2945	0.406	0.685	0.811
AHCYL1	4370	0.118	0.1937	0.609	0.542	0.708
MRPS23	3310	0.117	0.1758	0.664	0.506	0.677
C10orf2	1480	0.117	0.2152	0.544	0.586	0.74
L2HGDH	709	0.116	0.1386	0.839	0.401	0.586
LYRM2	2250	0.113	0.1653	0.685	0.493	0.666
C7orf55	44.3	0.112	0.1944	0.575	0.565	0.724
PET117	67.5	0.11	0.1505	0.728	0.466	0.644
TOP3A	3030	0.11	0.1766	0.622	0.534	0.701
AGK	1880	0.108	0.138	0.785	0.433	0.615
COX19	868	0.108	0.1582	0.684	0.494	0.667
ATAD3A	1410	0.108	0.2539	0.426	0.67	0.8
TSPO	1720	0.106	0.4939	0.214	0.831	0.903
CHPT1	1080	0.104	0.2467	0.423	0.672	0.802
MRPL19	2980	0.103	0.1403	0.731	0.465	0.642
NDUFB1	1040	0.103	0.1722	0.599	0.549	0.713
TIMM17B	1480	0.102	0.1567	0.649	0.517	0.686
COX10	781	0.102	0.1605	0.636	0.525	0.693

COX4I1	6050	0.1	0.1648	0.606	0.544	0.709
TRMT61B	1190	0.0998	0.1476	0.676	0.499	0.671
PDHX	1780	0.0995	0.1098	0.907	0.365	0.551
TOMM7	3080	0.0992	0.1943	0.511	0.609	0.757
NDUFS1	6610	0.0976	0.1311	0.745	0.457	0.635
HSD17B4	4330	0.0969	0.1323	0.732	0.464	0.641
BPHL	769	0.0969	0.1646	0.589	0.556	0.718
TRUB2	1750	0.0966	0.1563	0.618	0.537	0.703
CYB5R3	1750	0.0962	0.1854	0.519	0.604	0.753
GK	564	0.0959	0.1731	0.554	0.58	0.735
TIMM21	952	0.094	0.1725	0.545	0.586	0.739
NDUFS5	4490	0.0934	0.2445	0.382	0.703	0.822
UQCC1	1950	0.0924	0.1213	0.762	0.446	0.626
NDUFAF6	588	0.0915	0.1581	0.579	0.563	0.722
ALKBH7	712	0.0909	0.1943	0.468	0.64	0.781
FAHD2A	654	0.0904	0.1486	0.608	0.543	0.708
ACAD9	2050	0.0894	0.1469	0.608	0.543	0.708
CHCHD1	1020	0.0892	0.1681	0.531	0.596	0.747
SLC25A38	1320	0.089	0.177	0.503	0.615	0.762
HAGH	1500	0.0883	0.1187	0.744	0.457	0.635
SSBP1	3590	0.0865	0.1867	0.463	0.643	0.783
GHITM	6150	0.0862	0.185	0.466	0.641	0.781
GLS	6990	0.0834	0.1612	0.517	0.605	0.754
TBRG4	4540	0.0828	0.1727	0.479	0.632	0.775
CHCHD5	304	0.0823	0.1733	0.475	0.635	0.777
PREPL	7200	0.0822	0.2074	0.396	0.692	0.815
FAM162A	910	0.0776	0.2787	0.278	0.781	0.873
PGAM5	4630	0.0774	0.2477	0.312	0.755	0.856
IMMP1L	413	0.0767	0.1597	0.48	0.631	0.774
DLD	4010	0.0765	0.1849	0.414	0.679	0.807
HINT3	1350	0.0765	0.1909	0.401	0.689	0.813
CBR4	2020	0.0747	0.1854	0.403	0.687	0.812
ALKBH3	850	0.0739	0.1422	0.519	0.603	0.753
OGG1	803	0.0731	0.1867	0.392	0.695	0.817
ISCA1	1840	0.0728	0.1346	0.54	0.589	0.742
SLC25A41	15.3	0.0727	0.6438	0.113	0.91	0.951
DHRS7B	398	0.0724	0.2036	0.356	0.722	0.836
CAT	1380	0.0723	0.1925	0.376	0.707	0.825
DGUOK	2100	0.0722	0.1334	0.541	0.588	0.741
CYB5A	990	0.0721	0.2231	0.323	0.747	0.851
MRPS36	926	0.0714	0.1516	0.471	0.638	0.779
SDHC	1530	0.0704	0.3054	0.231	0.818	0.895
ATP5J	1900	0.07	0.1615	0.433	0.665	0.797
PMPCB	3500	0.0699	0.1691	0.413	0.679	0.807
SLC30A9	6600	0.0683	0.14	0.488	0.626	0.77
MRPS11	1080	0.0677	0.1513	0.447	0.655	0.79

FARS2	694	0.0669	0.1481	0.452	0.652	0.788
NIF3L1	1660	0.0668	0.1455	0.459	0.646	0.785
PARS2	360	0.0645	0.2202	0.293	0.77	0.866
DNAJC11	2140	0.0632	0.1436	0.44	0.66	0.793
FAHD1	1140	0.0627	0.1591	0.394	0.693	0.816
C20orf24	303	0.0616	0.2137	0.288	0.773	0.868
NDUFA3	869	0.0616	0.2274	0.271	0.786	0.877
PPOX	1070	0.0606	0.2097	0.289	0.773	0.868
TOMM6	2660	0.0595	0.1713	0.348	0.728	0.84
MRS2	1620	0.059	0.1667	0.354	0.724	0.837
POLG	2920	0.0588	0.1613	0.364	0.716	0.831
SPG7	2850	0.0584	0.1961	0.298	0.766	0.864
SUCLA2	2220	0.0551	0.1494	0.369	0.712	0.829
PDK3	1830	0.0546	0.2454	0.222	0.824	0.899
SLC25A36	3940	0.0539	0.1451	0.372	0.71	0.827
TRMU	1310	0.0533	0.1225	0.435	0.663	0.796
NME6	644	0.053	0.1713	0.309	0.757	0.858
PDF	135	0.0529	0.1889	0.28	0.779	0.872
05-Mar	1280	0.051	0.1948	0.262	0.794	0.881
MRPL35	2320	0.0504	0.1795	0.281	0.779	0.872
NBR1	5240	0.0497	0.2151	0.231	0.817	0.895
OXA1L	2300	0.0496	0.1601	0.31	0.757	0.858
NDUFB3	1580	0.0488	0.2217	0.22	0.826	0.9
TXNRD1	14300	0.0479	0.2518	0.19	0.849	0.916
ALDH5A1	985	0.0478	0.2069	0.231	0.817	0.895
IDH3G	1120	0.0474	0.1593	0.298	0.766	0.864
SLC25A5	6750	0.0448	0.2129	0.21	0.833	0.905
EXOG	644	0.0433	0.2592	0.167	0.867	0.927
NME3	930	0.043	0.2996	0.144	0.886	0.938
TIMM17A	3110	0.0426	0.2024	0.21	0.833	0.905
PGS1	1810	0.04	0.1229	0.325	0.745	0.85
PANK2	1920	0.0393	0.1353	0.291	0.771	0.868
PCCA	1000	0.0392	0.1432	0.273	0.785	0.876
PDSS2	587	0.0389	0.1396	0.279	0.78	0.873
DNAJC30	322	0.0383	0.1698	0.226	0.821	0.897
ATPAF1	1290	0.038	0.2073	0.184	0.854	0.919
CMC2	889	0.0375	0.2182	0.172	0.864	0.925
GRHPR	2320	0.0361	0.1535	0.235	0.814	0.893
NDUFC1	899	0.0352	0.194	0.181	0.856	0.92
MRPL51	2950	0.0328	0.2092	0.157	0.875	0.932
TIMM23	1600	0.0267	0.154	0.174	0.862	0.924
IDI1	3640	0.0265	0.1839	0.144	0.886	0.938
CISD3	576	0.0256	0.3082	0.0831	0.934	0.965
MUT	1600	0.0247	0.1593	0.155	0.877	0.933
MRPS12	820	0.0243	0.2299	0.106	0.916	0.955
POLG2	1010	0.0241	0.1739	0.138	0.89	0.941

KIAA0141	2710	0.0239	0.1669	0.143	0.886	0.938
COA6	637	0.0225	0.2466	0.0912	0.927	0.961
ACAD11	122	0.0219	0.2114	0.103	0.918	0.955
C3orf33	207	0.02	0.1447	0.138	0.89	0.941
FH	4250	0.0196	0.2265	0.0867	0.931	0.963
PDK1	2100	0.0176	0.3119	0.0564	0.955	0.976
PARK7	3340	0.0166	0.1879	0.0882	0.93	0.963
P4HB	13000	0.0165	0.2156	0.0767	0.939	0.968
GOLPH3	2600	0.0142	0.1344	0.105	0.916	0.955
NUDT6	187	0.0135	0.2132	0.0631	0.95	0.974
CLYBL	68.4	0.0107	0.5215	0.0206	0.984	0.992
COX7A2	4960	0.0104	0.1441	0.0721	0.943	0.97
GPX1	2970	0.0102	0.1969	0.0516	0.959	0.979
ACOX1	4270	0.00952	0.1702	0.0559	0.955	0.977
ALKBH1	605	0.00416	0.1349	0.0308	0.975	0.987
PITRM1	2660	0.00316	0.2269	0.0139	0.989	0.994
MRPS28	709	0.0031	0.1701	0.0182	0.985	0.992
TDRKH	1350	0.00192	0.2389	0.00804	0.994	0.996
MRPL55	1070	0.00177	0.1651	0.0107	0.991	0.996
ACSS3	19.7	-7.63	1.2569	-6.07	1.3e-09	2.28e-07
ALAS2	10.1	-7.06	2.7155	-2.6	0.00936	0.0469
COX7A1	5.67	-6.22	2.3889	-2.6	0.00925	0.0465
ABCA13	11.4	-5.38	1.0939	-4.92	8.67e-07	4.33e-05
AGXT	4.35	-5.06	2.0049	-2.52	0.0116	0.0545
AKR1B10	2.18	-4.97	2.2472	-2.21	0.0269	0.0988
MSRB3	546	-4.76	1.2442	-3.82	0.000132	0.00198
ACSM5	1.1	-4.12	3.0596	-1.35	0.178	0.351
RAB32	304	-4.05	1.0854	-3.73	0.000189	0.0026
PDK4	55.2	-3.73	1.3605	-2.74	0.00605	0.0342
TSTD1	121	-3.56	0.7427	-4.8	1.6e-06	7.02e-05
CYB5R2	51.2	-3.15	0.8639	-3.64	0.000268	0.0034
DNAJC15	311	-3.14	0.917	-3.43	0.000614	0.00635
SLC25A18	33.2	-3.08	0.8512	-3.62	0.000298	0.0037
MAOB	93.6	-2.92	1.287	-2.27	0.0233	0.0896
ALDH1L1	54.1	-2.9	0.7157	-4.06	4.99e-05	0.000941
NCEH1	724	-2.81	0.5087	-5.53	3.24e-08	3.32e-06
LDHD	23.6	-2.71	0.6348	-4.27	1.93e-05	0.000457
ECHDC2	37.4	-2.56	0.6695	-3.82	0.000134	0.002
SLC25A27	330	-2.53	0.5711	-4.43	9.43e-06	0.000275
MSRA	248	-2.51	0.5028	-5	5.7e-07	3.09e-05
STOM	1260	-2.46	0.9826	-2.51	0.0122	0.0564
ACCS	98.4	-2.41	1.1135	-2.17	0.0304	0.107
CYP27A1	130	-2.39	0.7066	-3.38	0.000723	0.00715
SLC16A11	11.8	-2.37	0.9674	-2.45	0.0142	0.0629
GLYAT	1.21	-2.37	1.1558	-2.05	0.0407	0.131
ACSL6	373	-2.33	0.6304	-3.7	0.000214	0.00285

AIFM3		77	-2.29	0.6029	-3.8	0.000146	0.00214
MGST1		277	-2.25	1.3115	-1.72	0.0857	0.216
SQRDL	87.8		-2.19	0.7478	-2.93	0.00334	0.0223
KMO	3.22		-2.09	0.7916	-2.65	0.00814	0.0425
CKMT2	3.73		-1.74	0.7709	-2.26	0.0238	0.0908
04-Sep		201	-1.73	0.5719	-3.03	0.00247	0.018
GATM		365	-1.63	0.7716	-2.12	0.0343	0.116
PINK1		1020	-1.58	0.3799	-4.15	3.27e-05	0.000686
AGMAT	26.5		-1.51	0.9692	-1.55	0.12	0.271
CASP8		132	-1.5	1.0656	-1.41	0.16	0.328
GPD2		1860	-1.4	0.6808	-2.06	0.0392	0.128
AIFM2		217	-1.39	0.559	-2.49	0.0128	0.0585
IFI27	29.9		-1.37	0.41	-3.35	0.000814	0.00784
ADHFE1	53.3		-1.36	0.3915	-3.48	0.000507	0.00551
ABAT		1880	-1.35	0.4464	-3.02	0.00255	0.0183
DHRS1		246	-1.32	0.2507	-5.28	1.27e-07	9.25e-06
NIPSNAP3B		182	-1.31	0.4536	-2.89	0.00385	0.0247
HSPB7	34.3		-1.28	0.8279	-1.55	0.122	0.274
ACACB		612	-1.27	0.2931	-4.34	1.44e-05	0.000368
C10orf10		99	-1.22	0.4986	-2.44	0.0146	0.0641
ABCD2		979	-1.22	0.6652	-1.84	0.0659	0.182
SLC25A21	7.77		-1.19	1.4616	-0.813	0.416	0.6
AMT		341	-1.13	0.4594	-2.46	0.014	0.0621
ECI2		1170	-1.11	0.4452	-2.48	0.013	0.0589
SUCLG2		843	-1.1	0.7354	-1.49	0.136	0.294
TMBIM4		709	-1.09	0.2098	-5.19	2.06e-07	1.37e-05
SDSL		106	-1.08	0.5829	-1.85	0.0649	0.18
CRAT		703	-1.07	0.342	-3.13	0.00174	0.0139
ACSF2		254	-1.06	0.6768	-1.56	0.118	0.268
NUDT19		616	-1.04	0.602	-1.73	0.0839	0.213
02-Mar		214	-1.01	0.6978	-1.45	0.146	0.309
PRSS35	63.9		-0.993	0.6546	-1.52	0.129	0.284
AASS		1270	-0.981	0.7882	-1.24	0.213	0.395
COQ10B		568	-0.974	0.2315	-4.21	2.6e-05	0.000574
CYP11A1	3.86		-0.971	1.1305	-0.859	0.39	0.576
EHHADH		388	-0.969	0.2994	-3.24	0.00121	0.0106
ALDH6A1		907	-0.951	0.2856	-3.33	0.00087	0.00825
CCDC109B		372	-0.951	0.7028	-1.35	0.176	0.349
GSTK1		1190	-0.945	0.2507	-3.77	0.000164	0.00234
RAB11FIP5		631	-0.945	0.3293	-2.87	0.00412	0.0259
COX6B2	14.2		-0.94	0.9146	-1.03	0.304	0.493
SLC25A43		219	-0.918	0.8757	-1.05	0.294	0.483
SLC25A48	6.53		-0.905	1.4951	-0.605	0.545	0.709
LACTB		444	-0.895	0.2897	-3.09	0.00201	0.0154
ABCD1		403	-0.893	0.2419	-3.69	0.000222	0.00293
DNAJC28	75.4		-0.889	0.2062	-4.31	1.62e-05	0.000402

HMGCL	351	-0.872	0.3448	-2.53	0.0114	0.0539
MUL1	1020	-0.871	0.168	-5.18	2.19e-07	1.45e-05
SLC22A4	59.2	-0.861	0.3924	-2.2	0.0282	0.102
ETHE1	416	-0.839	0.3232	-2.59	0.00947	0.0472
DDAH1	3060	-0.829	0.479	-1.73	0.0836	0.213
HSD17B8	1.36	-0.823	1.381	-0.596	0.551	0.714
CPT1C	2270	-0.811	0.4557	-1.78	0.0751	0.198
SFXN5	637	-0.787	0.2672	-2.95	0.00322	0.0217
SERHL2	15.7	-0.785	0.4427	-1.77	0.0762	0.2
TK2	733	-0.783	0.2575	-3.04	0.00235	0.0173
SPATA20	1720	-0.78	0.324	-2.41	0.016	0.0685
UCP1	0.658	-0.775	0.8204	-0.944	0.345	0.533
ME1	784	-0.771	0.7091	-1.09	0.277	0.465
SPR	412	-0.764	0.28	-2.73	0.00635	0.0355
CA5B	242	-0.745	0.2313	-3.22	0.00127	0.011
ECHDC3	197	-0.734	0.7029	-1.04	0.297	0.485
SYNJ2BP	3340	-0.726	0.1711	-4.24	2.19e-05	0.000504
CPS1	667	-0.703	0.7021	-1	0.317	0.506
COQ10A	778	-0.693	0.2219	-3.12	0.00179	0.0142
LYRM9	76.7	-0.685	0.464	-1.48	0.14	0.3
SLC25A42	264	-0.683	0.2785	-2.45	0.0142	0.063
NIPSNAP3A	979	-0.681	0.2059	-3.31	0.00094	0.00879
LAMC1	9790	-0.679	0.3081	-2.2	0.0275	0.1
ACADVL	4640	-0.678	0.2188	-3.1	0.00195	0.0151
THEM4	782	-0.672	0.5831	-1.15	0.249	0.436
SFXN3	1720	-0.656	0.3539	-1.85	0.0636	0.178
MFN1	3210	-0.652	0.1396	-4.67	3.01e-06	0.000114
SARDH	204	-0.651	0.4303	-1.51	0.13	0.286
NMNAT3	100	-0.651	1.4752	-0.441	0.659	0.793
NUCB2	1010	-0.646	0.2177	-2.97	0.003	0.0206
NUDT8	59.4	-0.645	0.2965	-2.18	0.0295	0.105
CROT	519	-0.637	0.2018	-3.16	0.00159	0.013
ACO1	1470	-0.629	0.2602	-2.42	0.0156	0.0672
PNPLA8	1800	-0.626	0.1538	-4.07	4.68e-05	0.000898
MGARP	74	-0.62	0.6165	-1.01	0.315	0.504
ACYP2	80.6	-0.618	0.3245	-1.9	0.0569	0.165
SUOX	593	-0.616	0.1397	-4.41	1.04e-05	0.000293
PDP1	2110	-0.616	0.2099	-2.93	0.00334	0.0223
PRELID2	384	-0.6	0.6135	-0.977	0.328	0.517
SLC30A6	2900	-0.598	0.1839	-3.25	0.00115	0.0102
GLS2	107	-0.578	0.6821	-0.848	0.397	0.581
ATAD3B	1330	-0.569	0.2339	-2.43	0.0149	0.065
CBR3	179	-0.562	0.7048	-0.798	0.425	0.608
NIT1	1060	-0.556	0.201	-2.77	0.00564	0.0325
COX20	1310	-0.554	0.2104	-2.63	0.00843	0.0435
SLC25A34	73.7	-0.551	0.3376	-1.63	0.103	0.245

CCDC90B		1900	-0.548	0.1262	-4.34	1.44e-05	0.000368
STX17		1320	-0.544	0.1369	-3.97	7.12e-05	0.00124
CEP89		653	-0.539	0.1986	-2.72	0.00659	0.0365
PAK7		518	-0.539	0.668	-0.807	0.42	0.603
CPOX		979	-0.534	0.177	-3.02	0.00256	0.0184
MTFR1L		1390	-0.533	0.181	-2.94	0.00326	0.0219
ABCB8		1220	-0.529	0.1471	-3.59	0.000327	0.00395
ABHD11		315	-0.529	0.237	-2.23	0.0255	0.0949
BAD		1050	-0.527	0.1539	-3.42	0.000623	0.0064
NLRX1		288	-0.522	0.6287	-0.83	0.407	0.591
MTCP1	67.8		-0.52	0.1901	-2.74	0.00618	0.0347
NFS1		838	-0.513	0.2267	-2.26	0.0237	0.0904
OBSCN		3090	-0.51	0.3895	-1.31	0.19	0.367
SIRT3		414	-0.508	0.2147	-2.37	0.018	0.0746
CPT1A		3320	-0.507	0.3892	-1.3	0.193	0.37
TXNDC12		1870	-0.498	0.2689	-1.85	0.0641	0.179
CMPK2		191	-0.487	0.4095	-1.19	0.234	0.418
HOGA1		123	-0.484	0.472	-1.03	0.305	0.494
CLIC4		9030	-0.481	0.3503	-1.37	0.17	0.34
MPV17		1310	-0.48	0.1843	-2.61	0.00914	0.0461
HARS2		2350	-0.475	0.1788	-2.65	0.00794	0.0417
PRODH		200	-0.472	0.5501	-0.858	0.391	0.576
ARMC10		1020	-0.471	0.1758	-2.68	0.00733	0.0394
ME3		656	-0.471	0.807	-0.583	0.56	0.72
SLC25A14		538	-0.462	0.1551	-2.98	0.00287	0.0199
SLC25A44		1620	-0.461	0.1949	-2.37	0.018	0.0746
ACOX3		587	-0.458	0.2164	-2.12	0.0344	0.117
HCCS		688	-0.454	0.1873	-2.42	0.0153	0.0664
BNIP3		2650	-0.452	0.2899	-1.56	0.119	0.27
FAM210A		951	-0.447	0.1531	-2.92	0.0035	0.0231
TRMT2B		633	-0.444	0.3161	-1.41	0.16	0.328
EFHD1	31.5		-0.443	0.9111	-0.486	0.627	0.771
PTRH1		216	-0.442	0.2163	-2.04	0.0409	0.132
HADHB		3890	-0.441	0.2004	-2.2	0.0279	0.101
NDUFAF7		1150	-0.44	0.1582	-2.78	0.00539	0.0315
PICK1		1060	-0.433	0.1322	-3.28	0.00105	0.00959
ADCK5		291	-0.43	0.1867	-2.3	0.0214	0.0839
HEMK1		1250	-0.429	0.1902	-2.26	0.024	0.0912
RDH14	88.4		-0.428	0.197	-2.17	0.0297	0.106
BCKDK		1070	-0.427	0.1575	-2.71	0.00678	0.0372
SURF1		886	-0.427	0.237	-1.8	0.0718	0.192
AMACR	31.5		-0.426	0.2375	-1.79	0.0729	0.194
TIMM10B		1570	-0.423	0.1805	-2.34	0.0191	0.0779
MOCS1		521	-0.42	0.1678	-2.5	0.0123	0.057
STOML1		493	-0.416	0.2419	-1.72	0.0854	0.216
MLYCD		385	-0.407	0.2124	-1.92	0.0555	0.163

FTH1		9450	-0.407	0.2774	-1.47	0.142	0.303
SLC25A29		2040	-0.404	0.2457	-1.65	0.0998	0.24
BNIP3L		4050	-0.39	0.237	-1.64	0.1	0.241
ALDH9A1		2160	-0.389	0.2172	-1.79	0.0731	0.195
ACP6		362	-0.386	0.37	-1.04	0.297	0.486
IARS2		7030	-0.383	0.2051	-1.87	0.0615	0.174
SLC25A35	71.1		-0.365	0.2678	-1.36	0.172	0.344
KIF1B		9210	-0.364	0.4254	-0.855	0.392	0.577
ADCK2		1190	-0.363	0.181	-2	0.0451	0.141
APEX2		799	-0.363	0.2055	-1.76	0.0777	0.202
NUDT13		117	-0.357	0.4965	-0.719	0.472	0.648
ATXN2		1630	-0.353	0.1577	-2.24	0.0254	0.0945
CHCHD7		341	-0.349	0.3035	-1.15	0.251	0.437
PDE12		1890	-0.337	0.156	-2.16	0.0309	0.108
COQ6		565	-0.335	0.1568	-2.14	0.0324	0.112
MRPL53		787	-0.333	0.1459	-2.28	0.0224	0.087
RAB35		2030	-0.332	0.1488	-2.23	0.0258	0.0957
MRPS26		2150	-0.332	0.1756	-1.89	0.0583	0.168
MICU2		1220	-0.332	0.1773	-1.87	0.0608	0.173
FTSJ2		1720	-0.331	0.189	-1.75	0.0803	0.207
APOA1BP		2400	-0.33	0.289	-1.14	0.254	0.441
PPM1K		1020	-0.329	0.428	-0.769	0.442	0.622
GUK1		4120	-0.328	0.206	-1.59	0.112	0.258
COX6B1		3780	-0.326	0.1766	-1.85	0.0646	0.179
SLC25A22		977	-0.322	0.1997	-1.61	0.107	0.251
PHYH		594	-0.322	0.2082	-1.55	0.122	0.274
FIS1		1750	-0.318	0.164	-1.94	0.0529	0.157
ISCA2		558	-0.316	0.1675	-1.89	0.0593	0.17
ECHS1		2220	-0.313	0.2082	-1.5	0.133	0.289
ADCK4		872	-0.311	0.1385	-2.24	0.0249	0.0934
FAM185A		434	-0.31	0.1565	-1.98	0.0472	0.146
SCO1		1570	-0.309	0.1767	-1.75	0.0805	0.207
C19orf70		1040	-0.309	0.1838	-1.68	0.0922	0.227
ACOT2		419	-0.309	0.5213	-0.593	0.553	0.716
MTRF1		482	-0.308	0.1981	-1.55	0.12	0.27
UCP3	66.7		-0.307	0.2534	-1.21	0.225	0.409
MPC2		1380	-0.299	0.2393	-1.25	0.211	0.393
ACADS		251	-0.299	0.3089	-0.969	0.333	0.522
TIMMDC1		2750	-0.298	0.1816	-1.64	0.101	0.242
MRPL17		1380	-0.296	0.1673	-1.77	0.0769	0.201
ATAD1		4350	-0.296	0.1677	-1.76	0.0777	0.202
VARS2	61.1		-0.295	0.9511	-0.31	0.756	0.858
MGST3		2060	-0.289	0.1711	-1.69	0.0907	0.225
OXCT1		2730	-0.28	0.3413	-0.821	0.412	0.595
NGRN		8010	-0.278	0.2231	-1.25	0.213	0.394
ACSL4		3410	-0.274	0.2389	-1.15	0.252	0.439

TMEM65	1580	-0.274	0.2483	-1.11	0.269	0.457
ACAD8	782	-0.27	0.1811	-1.49	0.136	0.293
MRPL43	1610	-0.269	0.1845	-1.46	0.145	0.308
ACAD10	1410	-0.268	0.1735	-1.55	0.122	0.274
BAK1	637	-0.266	0.2172	-1.23	0.22	0.404
PABPC5	152	-0.266	0.8965	-0.297	0.766	0.864
PRDX5	4210	-0.262	0.1055	-2.48	0.013	0.059
SCP2	1700	-0.259	0.2408	-1.08	0.282	0.47
LACE1	267	-0.257	0.1249	-2.06	0.0398	0.129
CMC4	122	-0.254	0.1684	-1.51	0.131	0.287
MPV17L2	423	-0.252	0.228	-1.1	0.27	0.458
DNAJC4	348	-0.252	0.2545	-0.99	0.322	0.511
GFM1	4000	-0.249	0.1533	-1.63	0.104	0.246
IDH3A	1280	-0.249	0.2089	-1.19	0.233	0.417
TUBB3	16500	-0.248	0.3539	-0.701	0.483	0.658
MRPL28	2120	-0.244	0.1544	-1.58	0.114	0.262
RHOT2	3140	-0.236	0.1251	-1.89	0.0589	0.169
ACAA1	570	-0.236	0.2349	-1.01	0.314	0.503
NDUFB5	2560	-0.234	0.1878	-1.24	0.213	0.395
CARKD	2280	-0.233	0.1297	-1.8	0.0722	0.193
COA5	1170	-0.232	0.1276	-1.82	0.069	0.187
RARS2	2480	-0.232	0.1586	-1.46	0.143	0.304
MSRB2	740	-0.232	0.2359	-0.983	0.325	0.514
NDUFA13	4800	-0.231	0.1526	-1.51	0.131	0.286
BCL2L2	997	-0.229	0.1092	-2.09	0.0362	0.121
ROMO1	1630	-0.227	0.1956	-1.16	0.246	0.433
FASTK	1850	-0.226	0.1341	-1.68	0.0925	0.228
SLC25A31	0.575	-0.225	1.0023	-0.224	0.823	0.898
RARS	4720	-0.224	0.2031	-1.1	0.271	0.459
MFN2	3980	-0.224	0.2429	-0.923	0.356	0.543
DHRS4	174	-0.222	0.2297	-0.967	0.333	0.522
SLC25A47	1.52	-0.222	0.647	-0.343	0.732	0.842
HDHD3	309	-0.22	0.1556	-1.41	0.158	0.325
BLOC1S1	299	-0.22	0.1621	-1.35	0.176	0.348
SETD9	233	-0.219	0.2373	-0.922	0.356	0.543
FUNDC1	345	-0.218	0.1713	-1.27	0.202	0.382
MTHFS	18.5	-0.216	0.2117	-1.02	0.307	0.496
RFK	1710	-0.215	0.2921	-0.736	0.462	0.64
ACOT9	988	-0.214	0.3644	-0.587	0.557	0.718
NADK2	1270	-0.211	0.1642	-1.28	0.199	0.379
LETM1	3580	-0.209	0.1603	-1.3	0.192	0.37
WBSCR16	1210	-0.209	0.1787	-1.17	0.241	0.426
HSDL1	1970	-0.208	0.241	-0.863	0.388	0.573
MTG2	1040	-0.206	0.1628	-1.26	0.206	0.387
MINOS1	573	-0.206	0.2171	-0.95	0.342	0.53
MTX2	1970	-0.204	0.1004	-2.03	0.042	0.134

NDUFAF3	1230	-0.204	0.206	-0.988	0.323	0.512
SCCPDH	2620	-0.202	0.2223	-0.907	0.365	0.551
MCUR1	2040	-0.197	0.1501	-1.31	0.19	0.367
METTL17	2300	-0.196	0.1569	-1.25	0.211	0.393
SDHAF2	1080	-0.192	0.1003	-1.92	0.0552	0.162
HK1	5650	-0.19	0.2515	-0.755	0.45	0.629
HIGD1A	2730	-0.188	0.2305	-0.815	0.415	0.599
MCU	924	-0.187	0.2194	-0.853	0.394	0.578
ACOT7	1670	-0.186	0.2292	-0.812	0.417	0.6
AKAP10	1580	-0.184	0.1545	-1.19	0.234	0.417
SDHAF1	529	-0.184	0.1669	-1.1	0.271	0.459
IDH3B	3000	-0.182	0.1441	-1.26	0.206	0.387
LIPT1	171	-0.182	0.187	-0.974	0.33	0.519
UQCRH	7240	-0.181	0.2559	-0.706	0.48	0.655
BCL2L1	1540	-0.179	0.1736	-1.03	0.301	0.49
NDUFS2	4390	-0.179	0.1821	-0.985	0.325	0.514
PTPN4	2700	-0.177	0.1708	-1.03	0.301	0.49
BAX	1390	-0.177	0.273	-0.647	0.518	0.687
ATPIF1	1440	-0.176	0.2098	-0.839	0.401	0.586
TFB2M	1220	-0.176	0.2267	-0.775	0.438	0.619
HEBP1	168	-0.175	0.4161	-0.421	0.673	0.803
C12orf10	1660	-0.174	0.135	-1.29	0.198	0.377
TFB1M	1070	-0.172	0.2601	-0.663	0.507	0.678
APOOL	946	-0.171	0.2476	-0.689	0.491	0.664
C15orf48 3.5		-0.169	0.5505	-0.306	0.759	0.86
RNASEH1	2170	-0.168	0.1672	-1.01	0.314	0.503
GDAP1	5050	-0.167	0.2831	-0.591	0.555	0.717
USMG5	3130	-0.166	0.1825	-0.908	0.364	0.551
SLC25A28	466	-0.166	0.23	-0.721	0.471	0.647
AUH	475	-0.164	0.1164	-1.41	0.16	0.328
SLC25A16	727	-0.162	0.1691	-0.959	0.337	0.526
MECR	653	-0.162	0.2214	-0.732	0.464	0.641
YME1L1	8080	-0.161	0.149	-1.08	0.281	0.469
PMPCA	2680	-0.161	0.169	-0.952	0.341	0.529
FASTKD5	1530	-0.16	0.1573	-1.02	0.309	0.498
MRPL34	1130	-0.159	0.1654	-0.964	0.335	0.524
OXR1	1370	-0.157	0.2602	-0.605	0.545	0.709
LONP2	4530	-0.156	0.1086	-1.43	0.152	0.317
MIPEP	616	-0.154	0.244	-0.632	0.527	0.695
SLC25A46	4210	-0.149	0.1247	-1.2	0.231	0.415
MRPS21	4240	-0.147	0.2037	-0.723	0.47	0.646
RMDN1	1570	-0.146	0.1618	-0.905	0.365	0.552
ATP10D	2490	-0.144	0.4079	-0.352	0.725	0.838
PYCR2	1890	-0.143	0.1946	-0.734	0.463	0.64
ETFB	1020	-0.143	0.3834	-0.374	0.708	0.826
MRPL20	1520	-0.14	0.2521	-0.557	0.578	0.733

AK4		1130	-0.139	0.4221	-0.328	0.743	0.849
PLGRKT		511	-0.136	0.1345	-1.01	0.311	0.5
MRPL52		1130	-0.135	0.143	-0.944	0.345	0.533
HIBADH		1190	-0.135	0.2265	-0.597	0.551	0.714
TMEM186		356	-0.133	0.1589	-0.834	0.404	0.588
COX5B		3850	-0.132	0.1265	-1.05	0.296	0.484
RPS18	26.2		-0.131	0.8091	-0.162	0.871	0.929
ARF5		2990	-0.13	0.1349	-0.964	0.335	0.524
KIAA0391		1830	-0.13	0.1357	-0.955	0.34	0.528
MRPS22		1460	-0.13	0.1836	-0.706	0.48	0.655
PDHB		2680	-0.126	0.1829	-0.69	0.49	0.664
SELO		907	-0.125	0.1347	-0.929	0.353	0.54
SPTLC2		3890	-0.125	0.1468	-0.852	0.394	0.579
TMEM143		164	-0.125	0.2051	-0.61	0.542	0.707
MTCH1		6070	-0.124	0.1412	-0.876	0.381	0.567
PEX11B		780	-0.124	0.1739	-0.713	0.476	0.651
MRPL41		1260	-0.124	0.227	-0.548	0.584	0.738
ISCU		2190	-0.123	0.1692	-0.727	0.467	0.644
C15orf40		577	-0.123	0.203	-0.605	0.545	0.709
AKR7A2		1230	-0.123	0.2393	-0.514	0.608	0.756
MTG1		1200	-0.122	0.2242	-0.544	0.586	0.74
ASAH2	12.6		-0.122	0.318	-0.383	0.702	0.821
AURKAIP1		2240	-0.12	0.2512	-0.477	0.633	0.776
CPT1B	30.8		-0.12	0.3254	-0.368	0.713	0.829
PARL		1430	-0.119	0.1511	-0.787	0.431	0.614
C16orf91		599	-0.118	0.1209	-0.975	0.329	0.518
SLC25A24		2910	-0.118	0.4458	-0.266	0.791	0.879
MTRF1L		930	-0.115	0.2287	-0.502	0.616	0.762
ATP5I		2620	-0.111	0.1433	-0.774	0.439	0.619
TUFM		6800	-0.109	0.1269	-0.855	0.392	0.577
SLC25A12		1170	-0.108	0.2311	-0.468	0.64	0.781
NSUN3		497	-0.107	0.1424	-0.752	0.452	0.631
NDUFA2		1390	-0.107	0.2139	-0.5	0.617	0.763
FKBP8		6230	-0.105	0.1122	-0.932	0.351	0.539
MAVS		4660	-0.104	0.155	-0.671	0.503	0.674
NDUFA1		1900	-0.104	0.1621	-0.639	0.523	0.692
DNLZ		343	-0.103	0.2297	-0.446	0.655	0.79
PDK2		865	-0.101	0.2079	-0.484	0.628	0.772
TSTD3		153	-0.0998	0.1936	-0.516	0.606	0.755
OTC	3.11		-0.0962	0.7579	-0.127	0.899	0.945
HSDL2		1290	-0.0961	0.2579	-0.373	0.709	0.827
RDH13		342	-0.0961	0.322	-0.298	0.765	0.864
TOMM40L		1010	-0.0947	0.198	-0.478	0.633	0.775
TCHP		1230	-0.0943	0.1352	-0.698	0.485	0.659
MALSU1		873	-0.0931	0.1781	-0.522	0.601	0.751
HIBCH		1080	-0.0905	0.143	-0.633	0.527	0.695

OPA3		886	-0.09	0.1929	-0.467	0.641	0.781
DBT		1390	-0.0892	0.2027	-0.44	0.66	0.794
SLC25A10		1560	-0.0872	0.2616	-0.333	0.739	0.847
UQCRQ		3930	-0.0859	0.1705	-0.504	0.614	0.761
PDPR		2980	-0.0854	0.1471	-0.581	0.562	0.721
PISD		1260	-0.0842	0.1633	-0.516	0.606	0.755
ANGEL2		2980	-0.0828	0.1362	-0.608	0.543	0.708
TXNRD2		619	-0.0814	0.1701	-0.478	0.633	0.775
CRYZ		953	-0.0805	0.3997	-0.201	0.84	0.91
HTRA2		1530	-0.0798	0.1107	-0.72	0.471	0.648
MDH1		6380	-0.0797	0.1219	-0.654	0.513	0.682
TRIT1		1160	-0.079	0.2545	-0.31	0.756	0.857
ABCB6		2310	-0.0784	0.1691	-0.464	0.643	0.782
MUTYH		706	-0.0773	0.2329	-0.332	0.74	0.847
C8orf82		781	-0.0772	0.2098	-0.368	0.713	0.83
DLST		2950	-0.0749	0.1361	-0.551	0.582	0.736
DNM1L		7770	-0.0719	0.1548	-0.465	0.642	0.782
ABCD3		2950	-0.0713	0.2061	-0.346	0.729	0.841
SLC25A20		562	-0.0703	0.3688	-0.191	0.849	0.916
ZADH2		2090	-0.0697	0.221	-0.316	0.752	0.855
COX6A1		6430	-0.0686	0.1675	-0.41	0.682	0.809
ISOC2		1010	-0.0677	0.2504	-0.27	0.787	0.877
SLC25A26		690	-0.0673	0.162	-0.415	0.678	0.806
SLC25A4		1830	-0.0671	0.2025	-0.331	0.741	0.847
TST		363	-0.0638	0.2052	-0.311	0.756	0.857
HINT2		636	-0.0623	0.1784	-0.349	0.727	0.839
APOPT1		705	-0.059	0.1588	-0.371	0.71	0.828
SIRT5		725	-0.0586	0.1653	-0.354	0.723	0.837
GSTO1		1930	-0.0585	0.2799	-0.209	0.834	0.906
COX16		101	-0.0577	0.1797	-0.321	0.748	0.852
OAT		3480	-0.0569	0.2137	-0.266	0.79	0.879
SLC25A40		1060	-0.0568	0.1604	-0.354	0.723	0.837
APOO		564	-0.0566	0.1543	-0.367	0.714	0.83
AFG3L2		3640	-0.0554	0.1629	-0.34	0.734	0.843
SLC25A51		1100	-0.0547	0.1289	-0.424	0.671	0.801
MRPS18B	0.54		-0.0544	0.8458	-0.0643	0.949	0.973
SDHB		1830	-0.0523	0.2984	-0.175	0.861	0.923
METTL15		793	-0.0516	0.1315	-0.392	0.695	0.817
COX14		675	-0.0512	0.1617	-0.317	0.751	0.854
SNAP29		1020	-0.0507	0.1153	-0.439	0.66	0.794
BDH1		639	-0.0477	0.2736	-0.174	0.862	0.924
ACADSB		821	-0.0474	0.2104	-0.225	0.822	0.897
ACADL	38.6		-0.0458	0.8878	-0.0516	0.959	0.979
NDUFV2		2320	-0.0455	0.2063	-0.22	0.826	0.9
IDH1		4370	-0.0451	0.2194	-0.206	0.837	0.907
SLC25A1		2580	-0.0449	0.1682	-0.267	0.79	0.879

ATP5E	70.4	-0.0434	0.2053	-0.211	0.833	0.905
C6orf203	477	-0.0417	0.1027	-0.406	0.685	0.811
GLRX2	421	-0.0411	0.199	-0.207	0.836	0.907
TOMM70A	7660	-0.0399	0.13	-0.307	0.759	0.859
FPGS	1520	-0.0398	0.1327	-0.3	0.764	0.863
SLC25A30	455	-0.0398	0.2248	-0.177	0.859	0.922
CPT2	901	-0.0383	0.2875	-0.133	0.894	0.943
CCDC51	755	-0.0361	0.1741	-0.207	0.836	0.907
COX4I2	35.3	-0.0357	0.9143	-0.039	0.969	0.984
TMLHE	499	-0.0351	0.142	-0.247	0.805	0.888
TMEM14C	2630	-0.0347	0.1478	-0.234	0.815	0.893
MRPL24	1890	-0.0344	0.257	-0.134	0.894	0.943
MMAB	1570	-0.0342	0.1706	-0.2	0.841	0.91
ADCK1	247	-0.0338	0.2141	-0.158	0.874	0.932
TIMM8A	612	-0.0336	0.1659	-0.202	0.84	0.909
OGDH	3330	-0.0327	0.149	-0.219	0.827	0.901
OXLD1	1210	-0.0326	0.1971	-0.165	0.869	0.928
COA1	3150	-0.0307	0.1872	-0.164	0.87	0.928
CARS2	1730	-0.0304	0.1215	-0.25	0.803	0.887
PTPMT1	1380	-0.0287	0.267	-0.107	0.914	0.954
C21orf33	2570	-0.0273	0.1266	-0.216	0.829	0.903
PRELID1	4290	-0.0257	0.2403	-0.107	0.915	0.954
NIT2	1310	-0.0233	0.1611	-0.145	0.885	0.938
NUBPL	709	-0.0226	0.136	-0.166	0.868	0.928
COQ4	987	-0.0224	0.133	-0.168	0.867	0.927
TCAIM	888	-0.0223	0.2102	-0.106	0.916	0.954
VWA8	2170	-0.0209	0.1501	-0.139	0.889	0.94
DBI	2110	-0.02	0.1693	-0.118	0.906	0.949
COQ9	1830	-0.0185	0.1483	-0.124	0.901	0.946
TCIRG1	301	-0.0181	0.5129	-0.0354	0.972	0.985
TIMM22	750	-0.0177	0.1753	-0.101	0.919	0.956
FDXR	753	-0.017	0.2318	-0.0733	0.942	0.97
MRPS33	1070	-0.0165	0.1609	-0.103	0.918	0.956
TPI1	23800	-0.0165	0.2138	-0.0769	0.939	0.968
NDUFB7	2250	-0.0161	0.126	-0.127	0.899	0.945
NT5C	1600	-0.015	0.1696	-0.0882	0.93	0.963
MRPL47	1920	-0.012	0.1952	-0.0613	0.951	0.974
TRMT10C	1810	-0.0107	0.1872	-0.0571	0.954	0.976
HSD17B10	2460	-0.0103	0.215	-0.048	0.962	0.98
NDUFB2	2030	-0.0102	0.1841	-0.0552	0.956	0.977
BCAT2	1260	-0.00899	0.2046	-0.044	0.965	0.982
ECH1	1150	-0.00894	0.1918	-0.0466	0.963	0.981
MRPS16	4750	-0.00847	0.1311	-0.0646	0.948	0.973
PSMA6	4910	-0.00722	0.1563	-0.0462	0.963	0.981
ATP5SL	1220	-0.00658	0.179	-0.0368	0.971	0.984
MTIF3	674	-0.00592	0.1528	-0.0387	0.969	0.984

RDH11	4120	-0.00572	0.1189	-0.0481	0.962	0.98
SLC25A13	3930	-0.00391	0.2811	-0.0139	0.989	0.994
MICU1	2200	-0.0032	0.1637	-0.0195	0.984	0.992
MTHFD1L	2360	1	0.2808	3.57	0.000355	0.00422
SHMT2	6730	1	0.2843	3.52	0.000434	0.00492
DMPK	769	-1	0.2417	-4.14	3.43e-05	0.00071
HTATIP2	125	-3	1.4292	-2.1	0.036	0.121
