# Supplementary material for: Iron Chelator VLX600 Inhibits Mitochondrial Respiration and Promotes Sensitization of Neuroblastoma Cells in Nutrition-Restricted Conditions
Source: Cancers (Basel). 2022 Jun 30;14(13):3225. doi: 10.3390/cancers14133225 (PMC9264775; doi:10.3390/cancers14133225)
Supplement: Supplementary file 1 [file cancers-14-03225-s001.zip › Supplementary Figure S1.pdf]

Supplementary Figure S1: Sk-N-BE(2) cells cannot form neat spheroids

Sk-N-BE(2) spheroid

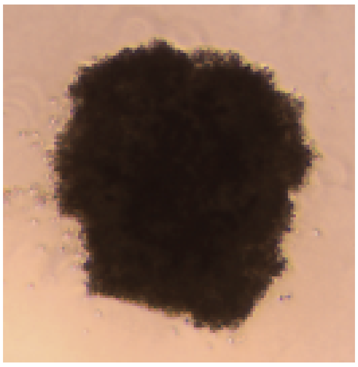

Sk-N-BE2 cells cannot form neat spheroids for the following spheroid diameter measurement, thus we only porforemd the spheroid experimnet on IMR-32 cells
