# Supplementary material for: Iron Chelator VLX600 Inhibits Mitochondrial Respiration and Promotes Sensitization of Neuroblastoma Cells in Nutrition-Restricted Conditions
Source: Cancers (Basel). 2022 Jun 30;14(13):3225. doi: 10.3390/cancers14133225 (PMC9264775; doi:10.3390/cancers14133225)
Supplement: Supplementary file 1 [file cancers-14-03225-s001.zip › Supplementary Figure S2.pdf]

Supplementary Figure S2: Combination effect of VLX600 and Etoposide on both IMR-32 and Sk-N-BE(2) cells

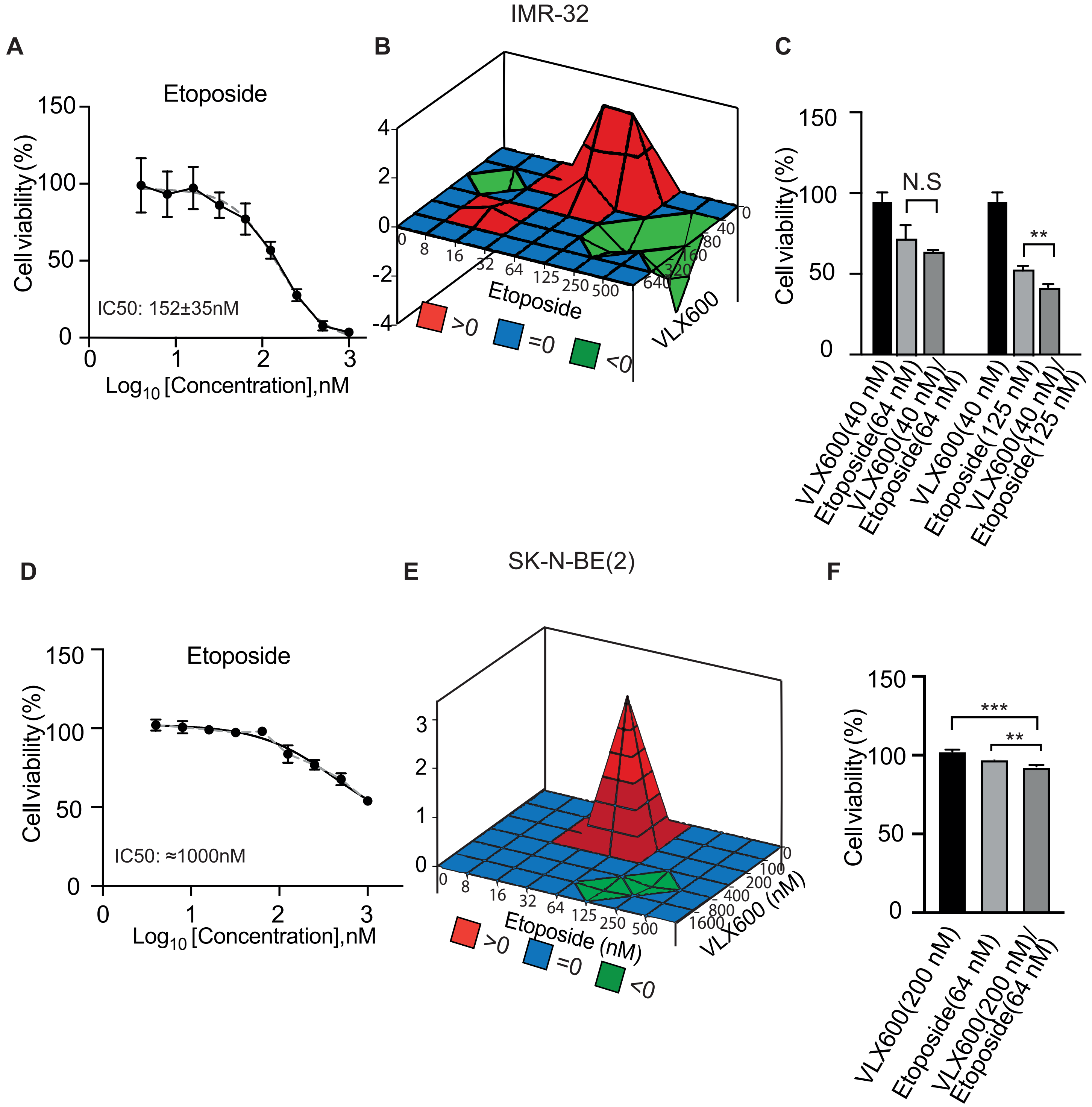

A. Dose-dependent sensitivity of MYCN-amplified IMR-32 neuroblastoma cells to etoposide (72hour treatment). B. Synergy plots generated by the MacSynergy™ II software, reflecting that there is a synergetic effect at the area of VLX600 40nM and etoposide 64nM or 125nM on IMR-32 cells. C. Statistical analysis on the cell viability data at VLX600 40nM and etoposide 64nM or 125nM on IMR-32 cells. Only the combination of 40nM VLX600 and 125nM etoposide generate a significant synergetic effect. D. Dose-dependent sensitivity of MYCN-amplified neuroblastoma Sk-N-BE2 cells to etoposide. IC50 is close to 1000nM. E. Synergy plots generated by the MacSynergy™ II software, reflecting that there is a synergetic effect at the area of VLX600 200nM and etoposide 64nM on Sk-N-BE2 cells. F. Statistical analysis on the cell viability data at VLX600 200nM and etoposide 64nM on Sk-N-BE2 cells. The combination shows a significant synergetic effect at a concentration only generating a minor effect on Sk-N-BE2 cells.
