# Supplementary material for: Iron Chelator VLX600 Inhibits Mitochondrial Respiration and Promotes Sensitization of Neuroblastoma Cells in Nutrition-Restricted Conditions
Source: Cancers (Basel). 2022 Jun 30;14(13):3225. doi: 10.3390/cancers14133225 (PMC9264775; doi:10.3390/cancers14133225)
Supplement: Supplementary file 1 [file cancers-14-03225-s001.zip › Supplementary Figure S3. Original WB image.pdf]

Figure 1H

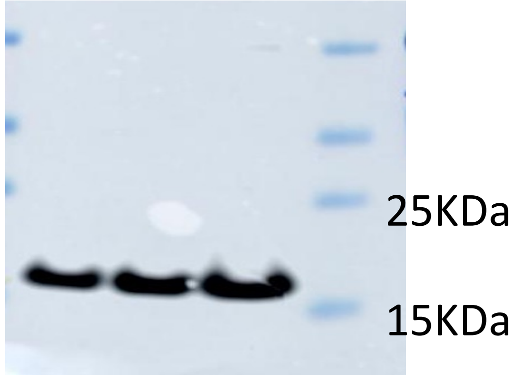

MT-COX4

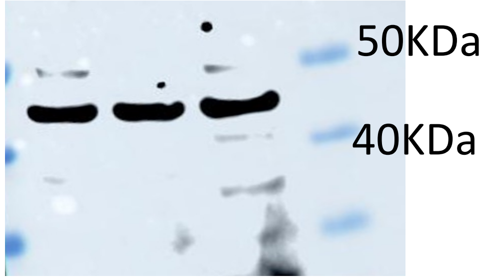

Actin

Figure 4

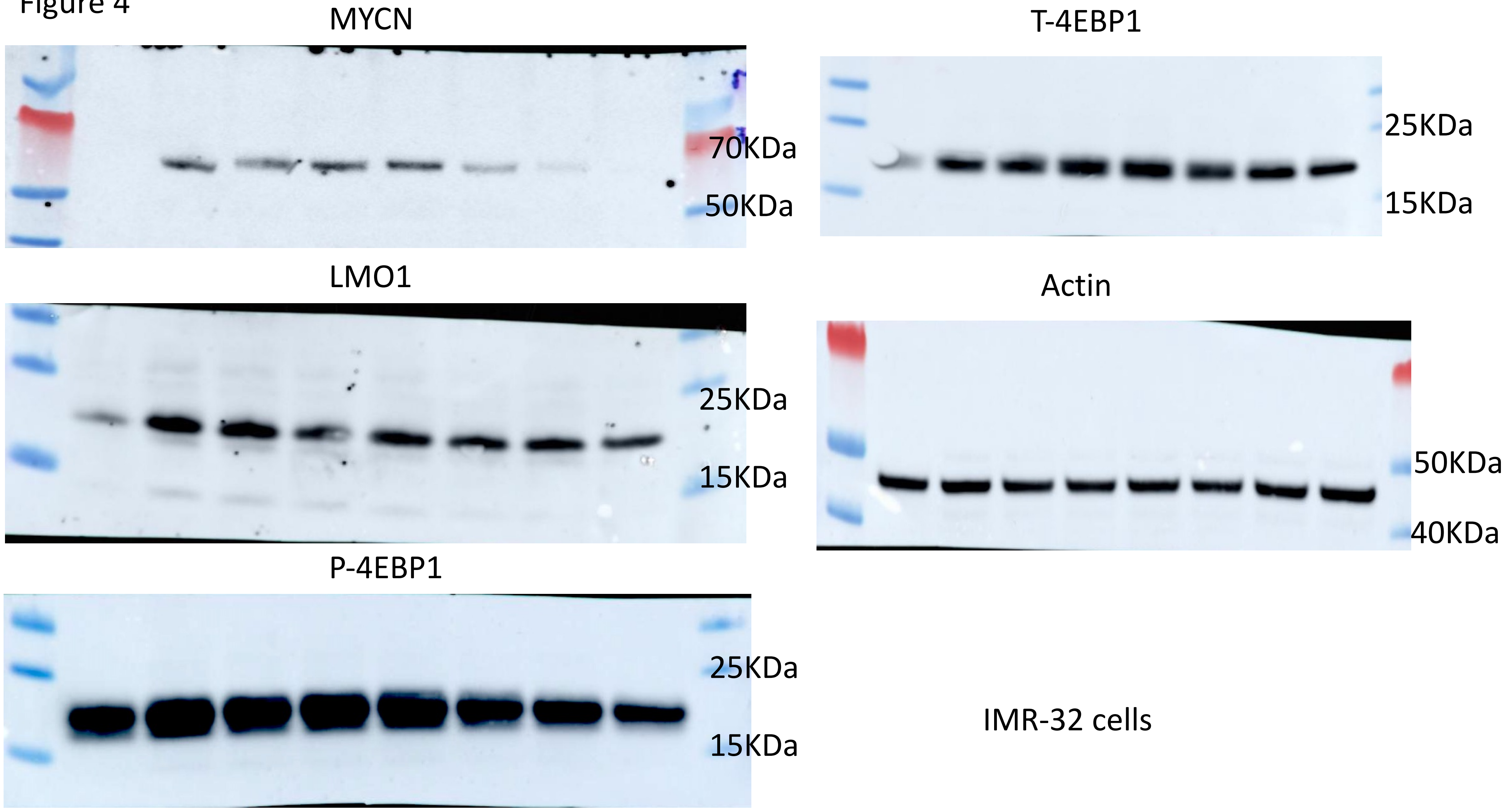
MYCN
70KDa
50KDa
T-4EBP1
25KDa
15KDa
LMO1
25KDa
15KDa
Actin
50KDa
40KDa
P-4EBP1
25KDa
15KDa
IMR-32 cells

Figure 4

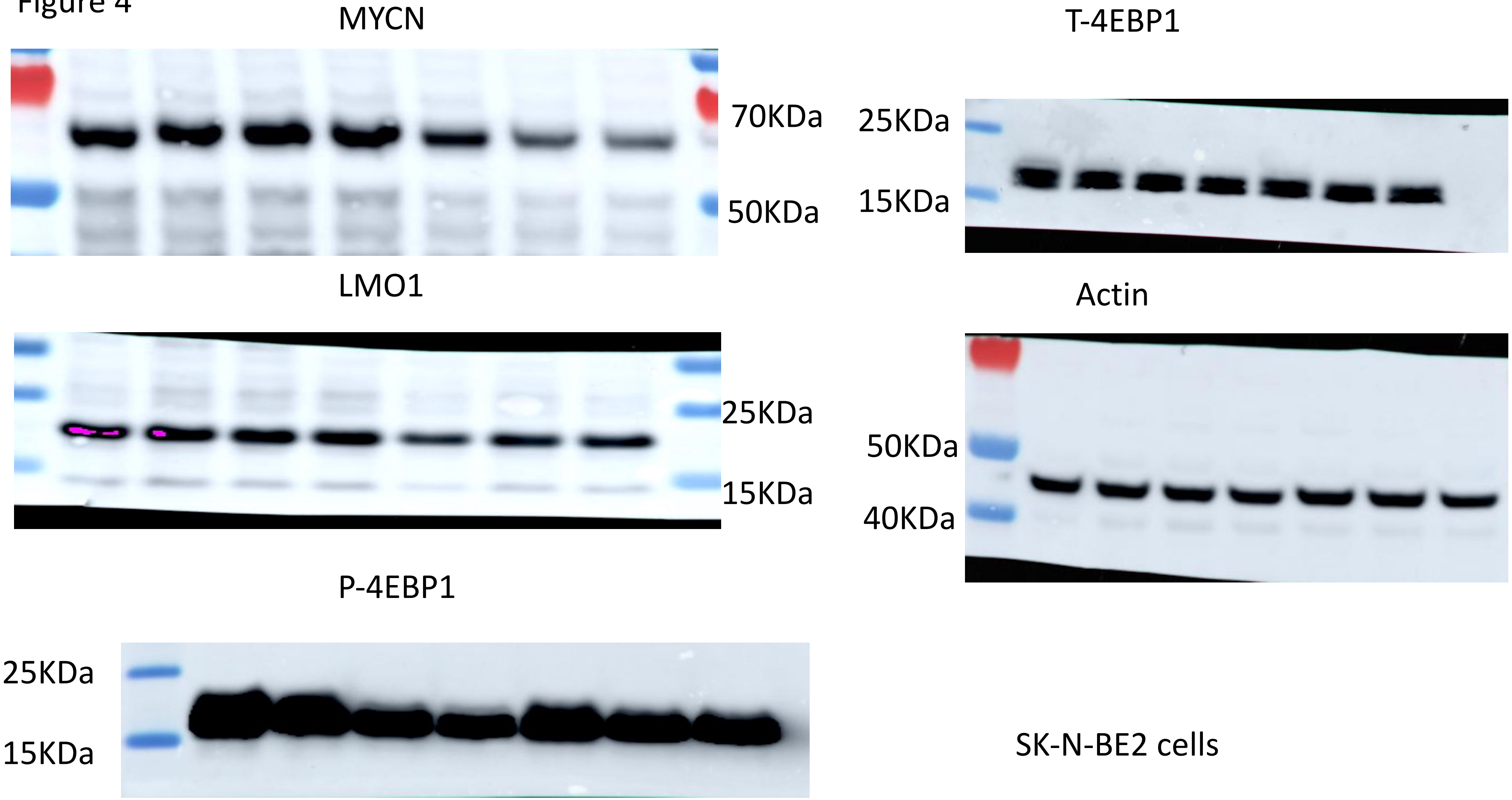
MYCN
70KDa
50KDa
T-4EBP1
25KDa
15KDa
LMO1
25KDa
15KDa
Actin
50KDa
40KDa
P-4EBP1
25KDa
15KDa
SK-N-BE2 cells

Figure 4D

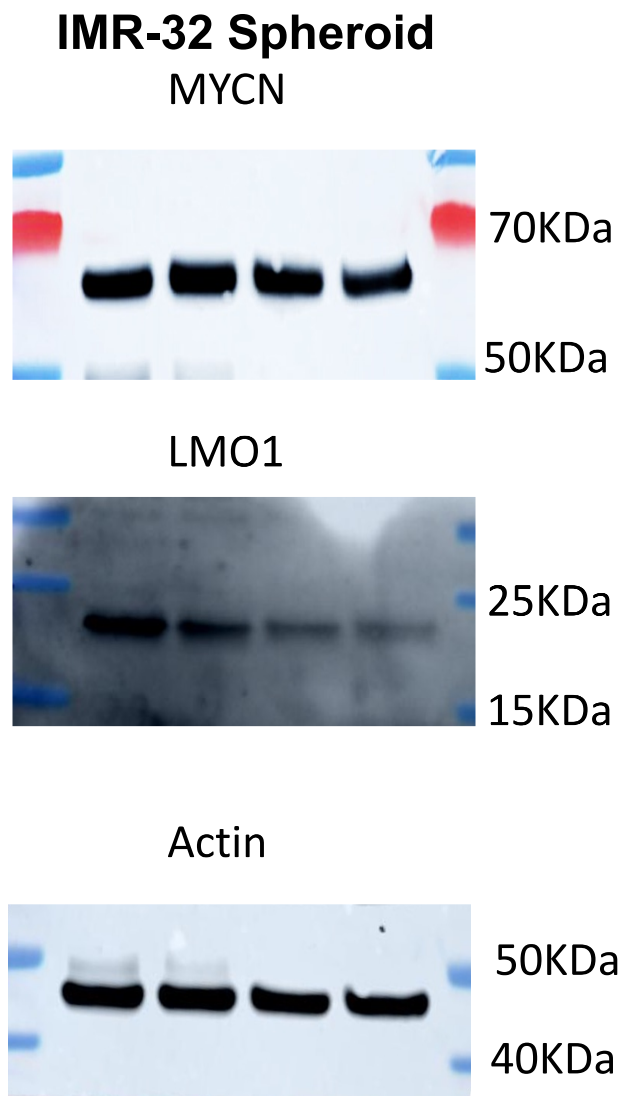

Figure 4E

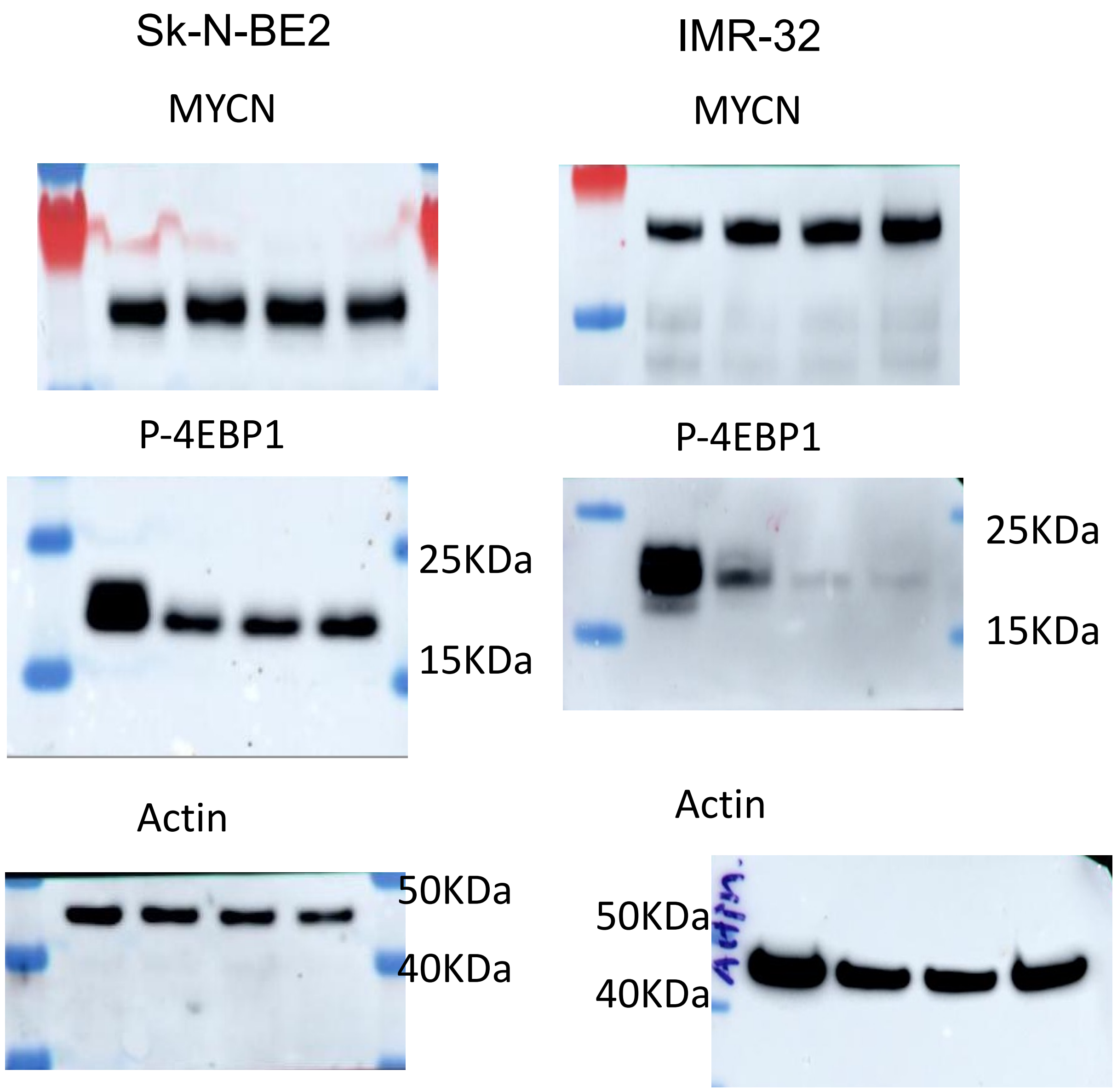

Figure 6

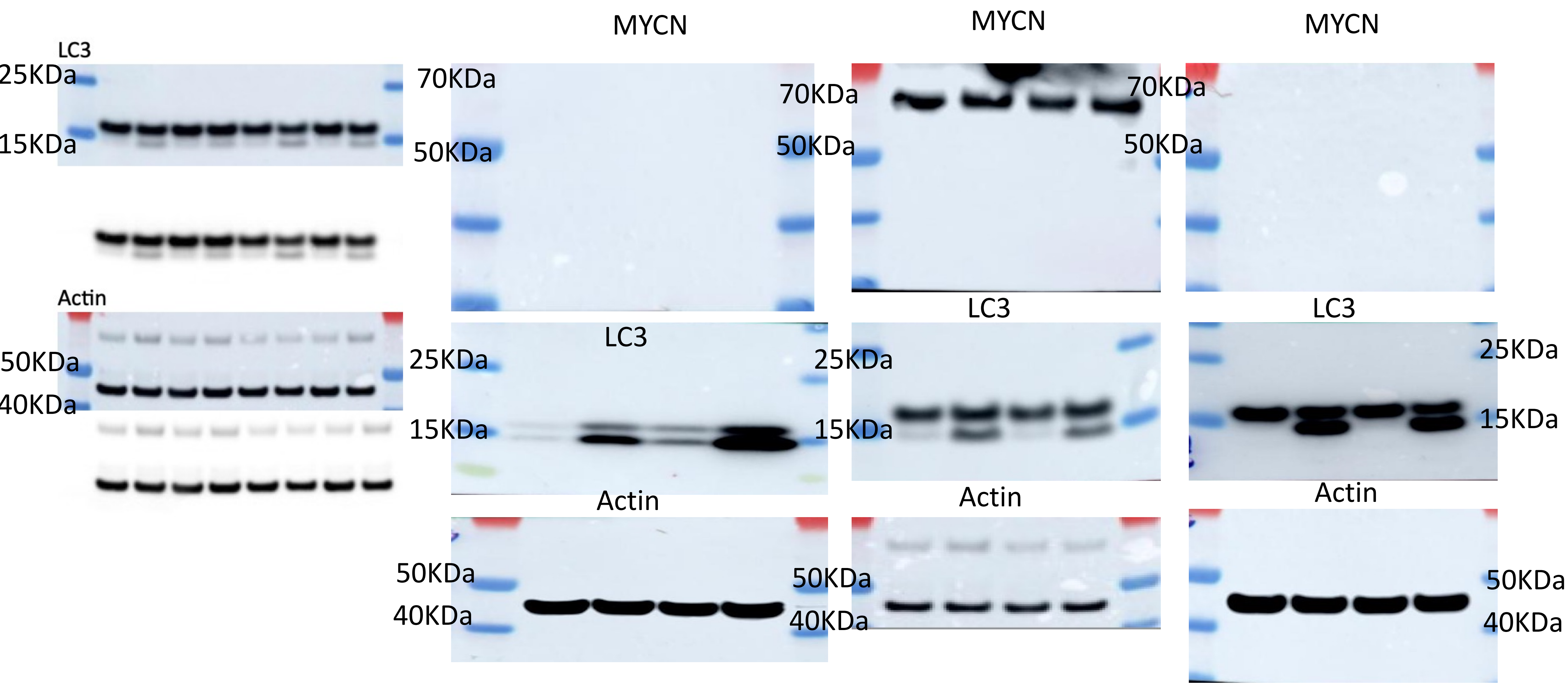

LC3
25KDa
15KDa
Actin
50KDa
40KDa
MYCN
70KDa
50KDa
LC3
25KDa
15KDa
Actin
50KDa
40KDa
MYCN
70KDa
50KDa
LC3
25KDa
15KDa
Actin
50KDa
40KDa
MYCN
70KDa
50KDa
LC3
25KDa
15KDa
Actin
50KDa
40KDa
